# Animal Models for Influenza Virus Pathogenesis and Transmission

**DOI:** 10.3390/v20801530

**Published:** 2010-07-27

**Authors:** Nicole M. Bouvier, Anice C. Lowen

**Affiliations:** 1 Division of Infectious Diseases, Mount Sinai School of Medicine, One Gustave L. Levy Place, New York, NY 10029, USA; E-Mail: nicole.bouvier@mssm.edu; 2 Department of Microbiology, Mount Sinai School of Medicine, One Gustave L. Levy Place, New York, NY 10029, USA

**Keywords:** influenza virus, transmission, pathogenicity, animal model, antiviral drug, influenza vaccine

## Abstract

Influenza virus infection of humans results in a respiratory disease that ranges in severity from sub-clinical infection to primary viral pneumonia that can result in death. The clinical effects of infection vary with the exposure history, age and immune status of the host, and also the virulence of the influenza strain. In humans, the virus is transmitted through either aerosol or contact-based transfer of infectious respiratory secretions. As is evidenced by most zoonotic influenza virus infections, not all strains that can infect humans are able to transmit from person-to-person. Animal models of influenza are essential to research efforts aimed at understanding the viral and host factors that contribute to the disease and transmission outcomes of influenza virus infection in humans. These models furthermore allow the pre-clinical testing of antiviral drugs and vaccines aimed at reducing morbidity and mortality in the population through amelioration of the virulence or transmissibility of influenza viruses. Mice, ferrets, guinea pigs, cotton rats, hamsters and macaques have all been used to study influenza viruses and therapeutics targeting them. Each model presents unique advantages and disadvantages, which will be discussed herein.

## 1. Influenza in the human host

### 1.1. Disease

Uncomplicated influenza is characterized by an acute onset of symptoms within one to two days of infection with influenza virus. Systemic symptoms, including fever and chills, headache, myalgia, lethargy, and anorexia, develop early in the course of illness. Fever generally ranges from 100 °F to 104 °F (38 °C to 40 °C), but may be as high as 106 °F (41 °C), with peak temperatures on the first day of symptoms and decreasing over three to eight days thereafter. While respiratory symptoms, including dry cough, pharyngeal pain, and nasal congestion and discharge, are also found, it is the presence of systemic symptoms that clinically differentiates influenza from other viral upper respiratory tract infections. Cough and sore throat may persist for several days after systemic symptoms abate [1].

Pulmonary complications of influenza virus infection include primary viral pneumonia and secondary bacterial pneumonia. Clinically, primary influenza viral pneumonia begins like typical uncomplicated influenza disease in the upper respiratory tract, but the acute illness rapidly progresses with signs and symptoms of lower respiratory tract disease, including cough, dyspnea, and hypoxemia. Secondary bacterial pneumonia follows a typical influenza illness; after an initial improvement lasting four to 14 days, recrudescence of fever, dyspnea, and cough with sputum signals the onset of a bacterial pneumonia [1].

In human influenza during interpandemic years, influenza virus infection is mainly confined to the upper respiratory tract, and primary viral pneumonia rarely occurs; when it does, patients tend to be older, with cardiovascular comorbidities. During pandemic years, including 1918, 1957, 1968, and 2009, the epidemiology of influenza disease has shifted, with younger populations disproportionately affected by lower respiratory tract disease and hospitalization [1–3].

### 1.2. Transmission

When considering influenza in humans, viral shedding is often used as a proxy measure of contagiousness. In a recent meta-analysis of volunteer challenge studies [4], shedding was found to begin within the first day after inoculation, peak on day 2, and cease by day 8 or 9 post-infection. Overall, only 66% of the experimentally inoculated patients developed disease and, although the available data was limited, shedding was detected in asymptomatic volunteers [5–7]. Average symptom scores peaked on day three post-infection, indicating that viral shedding precedes the development of disease by approximately one day.

Observational studies carried out during the 2009 H1N1 pandemic produced estimates of the secondary attack rate among household contacts in the range of 11–13%, with serial intervals estimated to be 2.4–2.9 days [8–10]. Most transmission events were reported to occur shortly before or after the onset of symptoms [8]. That a pre-symptomatic or asymptomatic person might act as an index case is also supported by the detection of influenza viruses in the normal exhaled breaths of infected individuals [11,12].

Specific host factors that may render some individuals more efficient spreaders of influenza virus than others have, for the most part, not been identified. Epidemiological observations as well as clinical data on the duration of viral shedding suggest that children (most likely with no prior exposure to influenza viruses) and immunocompromised individuals are good transmitters [13–18]. Thus, immune competence and history of exposure to influenza are most likely major factors affecting influenza virus transmission.

The mode of transmission of influenza virus has been examined to some extent, but uncertainty remains as to the relative importance of airborne, droplet, and contact-based spread. Observational studies of influenza outbreaks [reviewed in 19] suggest roles for both contact and airborne transmission. Furthermore, early work in humans showed that the infectious dose of influenza virus required to cause disease was lower when the inoculum was applied as an aerosol, as opposed to nasal droplets [20]. Recent reviews of the literature on this topic [21,22] have reached conflicting conclusions on the importance of small droplet aerosols, an issue which has significant implications for the infection control of influenza.

## 2. Animal models of influenza

Laboratory animal models are widely used in the preclinical evaluation of potential vaccines and antiviral compounds, to investigate the safety of the vaccine or compound and its efficacy in preventing or moderating infection, disease or secondary transmission. In selecting an animal model for such research, a number of essential factors must be considered. The animal must be susceptible to influenza virus infection and supportive of its replication, with a sensitive and specific “read-out” for viral infection, which is then altered in the presence of an antiviral compound with activity against the virus. Depending on the model species, read-outs can include clinical signs such as weight loss, lethargy, and pyrexia, or histopathological changes in or virus recovery from tissues such as nasopharynges or lung. Amelioration of these clinical, virological, or histopathological parameters in the presence of an investigational drug or vaccine suggests its antiviral efficacy in that animal model.

The animal model must also represent humans, in terms of similarity of clinical signs, histopathologic changes, virus growth kinetics, or transmission. Some animal models, like ferrets and guinea pigs, are naturally susceptible to infection by human influenza strains; others, like mice, require adaptation of the human virus to the species.

### 2.1. Mice (Mus musculus)

Mice are the most widely used animal model for influenza virus research. The reasons for this are largely practical ones: the mouse is a convenient model in terms of size, cost and husbandry requirements; and the availability of species-specific reagents, coupled with our ability to manipulate mice genetically, offers a system in which the host response to infection can be studied in depth. For preliminary assessments of drug or vaccine safety and efficacy, the mouse model is often the best choice. There are, however, a number of drawbacks of the model that make it unsuitable for addressing certain virological questions and can render data obtained in mice difficult to translate to the human situation.

#### 2.1.1. Susceptibility of mice to human and other influenza viruses

The susceptibility of mice to influenza viruses depends both on the strain of mouse and on the strain of influenza virus. The majority of influenza virus research in mice employs either BALB/C or C57BL/6 strains in conjunction with the lab adapted A/Puerto Rico/8/1934 (H1N1) [PR8] or A/WSN/1933 (H1N1) [WSN] influenza viruses.

Most inbred laboratory mice are highly susceptible to disease and death following intranasal infection with certain influenza viruses, including PR8 and WSN; in contrast, wild mice are resistant to even very high doses of the same viral strains [23–25]. The basis for this difference has been shown to be the lack of expression of a functional Mx1 protein, a critical antiviral factor [26], in most inbred laboratory mice. Indeed, the 50% lethal dose of PR8 virus in mice carrying a knocked-in *MX1* gene is >1000-fold higher than in the parental C57BL/6 strain [27], while the lethal doses of the highly virulent A/Viet Nam/1203/04 (H5N1) and 1918 pandemic strains are increased by >100-fold with the expression of Mx1 in BALB/C mice [28].

In addition, certain inbred strains have been found to be more prone to weight loss and death following influenza virus infection than others. In particular, DBA/2J and A/J mice were shown to be more susceptible to disease, even with viral isolates that were not adapted to mice, than the more commonly used BALB/C and C57BL/6 strains [29–31]. This finding allowed the use of DBA/2J mice for the study of 2009 pandemic influenza viruses without adapting these new strains to the mouse model [32–35]. Although increased pathology in DBA/2J mice has been correlated with a heightened inflammatory response [29,30], a causal relationship has not been established. Higher cytokine levels in mouse strains with greater susceptibility may be simply the result of increased viral replication relative to that seen in more resistant mice. It does seem clear that differences in host genetics account for the differing susceptibilities of DBA/2J and C57BL/6 mice to influenza viruses: gene mapping studies using recombinant inbred progeny of DBA/2J and C57BL/6 strains identified three loci associated with resistance to influenza virus induced pathology and indicated that host resistance is a complex, multigenic trait [30].

Even inbred mouse strains are resistant to disease following infection with most primary human influenza virus isolates [36–39]. For this reason, the mouse model is most often used with influenza virus strains that have previously been adapted through serial passage in this host. In particular, the early human isolates, PR8 and WSN, have become the prototype lab strains used in the mouse model. Laboratory mice are highly susceptible to infection with these viruses and severe disease or death is observed following administration of relatively low doses [40]. Due to the fact that it has been in use for several decades, a number of distinct lineages of PR8 virus (with a range of 50% mouse lethal doses) exist in various laboratories [27]. For studies requiring an H3N2 subtype influenza virus, X31, a reassortant virus carrying the HA and NA genes of A/Hong Kong/1/1968 (H3N2) in the background of PR8, is often used [40–47]. The virulence of X31 to mice is intermediate between that of wild-type human H3N2 isolates and PR8, in that a dose of approximately 10^6^ plaque forming units (PFU) of X31 is required to cause death in BALB/C mice [40,48]. As with the human-adapted influenza A viruses, influenza B viruses in general do not cause disease in mice. The mouse-adapted strain B/Lee/1940 does, however, bring about severe weight loss when a dose of 5 x 10^5^ PFU is administered intranasally to BALB/C mice [49].

Although mouse adapted strains are needed to model seasonal H1N1 and H3N2 virus infections, certain influenza viruses cause disease in mice without prior adaptation. These include the 1918 H1N1 pandemic strain [36], highly pathogenic avian influenza (HPAI) viruses of the H5N1 subtype [48,50–52], certain H7 subtype viruses [52–55], a subset of low pathogenic avian influenza viruses [56] and the 2009 H1N1 pandemic strains [39,57,58]. The susceptibilities of BALB/C or C57BL/6 mice to infection with and disease induced by these viruses are indicated by their 50% infectious (ID_50_) and 50% lethal doses (LD_50_), respectively (summarized in Table 1). The body weight loss and mortality that result from infection allow the mouse model to be used to assess the efficacy of treatments and vaccination regimens specifically against these viral strains. Thus, the FDA approved antiviral drugs rimantidine and amantadine have been shown to be effective against the 1918 pandemic strain in a mouse model [59] and similar studies have determined that drugs currently in use for the treatment of seasonal influenza are also effective against H5N1 strains [51,60–64]. A number of antiviral drugs in the preclinical stages of development have furthermore been tested in mice with a lethal H5N1 influenza virus challenge [65–72] and inactivated viral vaccines based on historical H1N1 isolates have recently been shown to provide protection to mice challenged with a lethal dose of the 2009 pandemic strain, A/Netherlands/602/2009 (H1N1) [73].

One important criterion for host susceptibility to influenza virus infection is the presence of the appropriate sialic acid receptors on epithelial cells of the target tissue. In general, human influenza viruses attach preferentially to sialic acid moieties with an α2,6 linkage to galactose [77,78], and such receptors are abundant in human upper airways [79,80]. α2,6 linked sialic acids are not, however, abundant in the mouse respiratory tract; instead, sialic acids with an α2,3 linkage predominate in the airways of mice [37,79]. The relative lack of human influenza virus receptors in mice may explain why human isolates do not replicate well in this species without prior adaptation. Indeed, an examination of virus attachment to respiratory tissues *ex vivo* indicated that, while avian influenza viruses bound throughout the murine upper and lower airways, human strains attached at low levels only to cells of the alveoli; by contrast, human influenza viruses bound strongly to human tracheal and bronchial tissues [81].

#### 2.1.2. Signs of disease in mice

When an appropriate pairing of viral and mouse strains are selected, the mouse model represents a convenient means of assessing influenza virus pathogenicity and its reduction through the use of vaccines and antiviral drugs. The signs of disease that develop and their severity depend upon the challenge dose administered. In most cases, however, a lethal dose is used resulting in a severe disease characterized by huddling; ruffled fur; lethargy; anorexia, which leads to weight loss; and death (euthanasia at a humane endpoint). Necropsy of mice with severe influenza reveals lung lesions characteristic of pneumonia, including pulmonary edema and inflammatory infiltrates [75,82–85]. Many HPAI viruses of the H5 and H7 subtypes spread beyond the mouse respiratory tract to other tissues including the brain, spleen, thymus, kidney, liver and heart [48,52,53,55,76,82]. This systemic spread is possible due to the presence of a multi-basic cleavage site in the viral hemagglutinin protein and results in the development of additional disease signs not seen with viruses confined to the respiratory tract, such as hind leg paralysis and mild encephalitis [86,87]. Although it does not carry a multi-basic cleavage site, the mouse adapted strain, WSN, also spreads to the brain and causes neurological symptoms when administered intranasally at high doses [74].

Several important differences exist between the manifestations of influenza in mice and humans. Unlike humans, mice do not develop fever following influenza virus infection; conversely, hypothermia has been reported [82,88–90]. In mice, viral replication and resultant tissue damage are concentrated in the lower respiratory tract rather than the upper airways [53,57,82,91]. Most likely as a consequence of this differing tropism, influenza in mice is generally more severe than what normally occurs in human hosts. In the case of HPAI viruses in mice, systemic spread is more pronounced than is seen in humans, where high viral titers are detected only in the respiratory tract [92]. Also, some H7 viruses are highly pathogenic in mouse but of low pathogenicity in humans; thus, even when mice are naturally susceptible to disease, virulence does not always correlate to that seen in humans [55]. Finally, neither coughing nor sneezing is observed in mice.

The parameters most commonly used to evaluate influenza viral pathogenicity in mice are body weight loss and mortality. In addition, viral titers, pathology scores, lung weights, oxygen saturation in the blood [93,94], and gross motor-activity levels [95] may be monitored. The main advantage of using the mouse model is that large numbers of animals may be used, allowing statistically robust data to be obtained easily. For this reason, initial *in vivo* safety and efficacy studies for antiviral drugs currently approved for use against influenza viruses were performed in the mouse model. The antiviral effects of the M2 ion channel inhibitors amantadine and rimantadine were first demonstrated *in vivo* in mice [96–99]. More recently, Mendel *et al*. demonstrated that treatment of mice with the neuraminidase inhibitor oseltamivir (Tamiflu®) enhanced survival and decreased viral load in the lung [100], while Sidwell and colleagues extended these findings to include effects of treatment on arterial oxygen saturation and lung consolidation [94]. Von Itzstein *et al*. employed the mouse model to show that zanamivir (Relenza®) treatment by the intranasal, but not the intraperitoneal, route reduced lung titers in influenza virus infected animals [101]. Finally, the most recently approved antiviral for influenza, peramivir, was first demonstrated to increase survival, reduce viral load, and inhibit consolidation of the lungs in a mouse challenge model [102–104].

#### 2.1.3. Transmission of influenza viruses in mice

Early studies on the transmission of influenza viruses from infected to naïve animals were performed in a mouse model [105–111]. Male Manor Farms (MF-1) or CFW mice were found to transmit influenza viruses by both aerosol and contact routes. The efficiency of transmission varied with the strain of influenza virus and ranged from 5% to 62.5% in this system. The influenza viruses used included mouse adapted H1N1 (PR8, NWS, A/CAM/46, A/FM/1) and H2N2 (A/Ann Arbor/6/1960 and A/Japan/305/1957) strains, as well as one H2N2 isolate that had not been serially passaged in mice. In general, H2N2 subtype viruses were found to transmit more readily than H1N1 strains, and the non-mouse-adapted H2N2 virus transmitted with 30% efficiency [108]. More recent attempts to model influenza virus transmission in BALB/C mice have been unsuccessful: direct contact transmission failed to occur when mice infected with high doses of the mouse adapted WSN strain, the 1918 pandemic virus, a highly pathogenic H5N1 isolate, human seasonal H1N1 or the 1968 H3N2 pandemic strains were co-caged with naïve mice [112]. The reason for the discrepancy between the work of Schulman and colleagues and more recent efforts to study influenza virus transmission in mice is unclear, but differences in mouse strains and husbandry practices seem likely to play a role (e.g. bacterial co-infection may have been more prevalent in the vivaria of the 1960s). It is also of note that the studies reported in [112] did not include an H2N2 subtype influenza virus.

### 2.2. Ferrets (Mustela putorius)

The ferret has been used to model human influenza virus infection since the virus was first isolated from humans in the 1930s. The model has been validated through years of experience, and the ferret is thought to most accurately represent human influenza disease. Ferrets are susceptible to a wide variety of human influenza virus isolates without prior adaptation to the species, and, like humans, they demonstrate a primarily upper respiratory tract infection with seasonal influenza strains. The main disadvantages of ferrets as an experimental model are their size, expense, and husbandry requirements, which make the model inaccessible to some researchers.

#### 2.2.1. Susceptibility of ferrets to human and other influenza viruses

Ferrets, like mice, have been used in studies of influenza virus pathogenesis since the initial isolation of influenza viruses from swine and humans in the early 1930s [113–118]. In 1933, Smith, Andrewes, and Laidlaw first isolated influenza virus from humans by intranasally inoculating ferrets with filtered throat washings from patients with influenza. Three days post-inoculation, animals developed signs consistent with human influenza disease, including fever, nasal discharge, lethargy, weakness, and anorexia; after a variable period of illness, from three to 10 days, the ferrets recovered uneventfully [119].

Unlike mice, ferrets are naturally susceptible to unadapted human influenza virus isolates, including influenza A subtypes H1N1 (both pre-2009 seasonal [120–122] and 2009 swine-origin pandemic [39,123,124] strains), H2N2 [66], H3N2 [125–128], and H5N1 [76,126,129–131] subtypes and influenza B viruses [132–134]. They can also be infected with influenza A viruses isolated from other species, including birds [135,136] and swine [116].

#### 2.2.2. Signs of disease in ferrets

Early experiments demonstrated that ferrets inoculated with human influenza virus exhibit overt disease, including fever, nasal congestion and discharge, anorexia, lethargy, and sneezing [119,137]; this symptomatology is similar to influenza in humans [1]. Although sneezing can occur in all upper respiratory tract infections in humans, it is more frequently associated with the common cold (caused by mainly rhino- and coronaviruses) than with influenza [138–140], whereas sneezing is a prominent feature of ferret influenza [141]. Ferrets have an exquisite sneeze reflex; indeed, early experimenters found that intranasal inoculation required anesthesia so that intranasal instillation of influenza virus did not induce expulsion of the inoculum itself [116].

Like humans, ferrets inoculated with human influenza viruses demonstrate a primarily upper respiratory tract infection, with tissues lower in the respiratory tract decreasingly affected [142,143]. Some strains of influenza virus have been noted to be more pathogenic in the ferret lower respiratory tract, including the reconstructed 1918 pandemic virus [144]; A/swine/Iowa/1930 (H1N1), a virus antigenically and genetically similar to the 1918 pandemic strain [116]; and H5N1 avian viruses isolated from humans [76,131]. Virus histochemistry studies have demonstrated that seasonal human strains of H3N2 and H1N1 subtypes attach predominantly in the upper respiratory tract (trachea and bronchi) of both humans and ferrets. In contrast, an H5N1 avian influenza virus attached relatively more abundantly in the lower respiratory tract (alveoli and bronchioles) of both species. These studies suggest that different tissue tropism of influenza viruses of human and avian origin may explain, in part, their different patterns of disease, and highlight a similarity between human and ferret influenza virus infections [145,146].

Because they show clinical symptoms, ferrets are often used to test antiviral agents for efficacy in preventing influenza disease. All three clinically available neuraminidase inhibitors – oseltamivir, zanamivir, and peramivir – have shown efficacy in the ferret model, as measured by a reduction of influenza virus shedding in the nares, a decrease in febrile response or other inflammatory markers, or, in the case of high-pathogenicity avian viruses, improved survival of ferrets inoculated with a lethal dose [100,101,147,148]. A study by Boltz *et al*. demonstrated that oseltamivir, when given prophylactically prior to infection with the highly pathogenic avian influenza virus A/Vietnam/1203/04 (H5N1), can mitigate weight loss and fever, reduce viral shedding in the upper respiratory tract, and prevent death in ferrets [129]. Similar studies have been done with experimental antivirals, such as DAS181 (Fludase®), a recombinant fusion protein that blocks influenza virus infection by eliminating cell surface viral receptors. In ferrets, a related construct DAS178, when administered from two days prior to five days after inoculation with influenza A/Bayern/7/95 (H1N1)-like virus, was shown to prevent infection in three of 12 ferrets and to alter the kinetics of nasal viral shedding in the remainder; all animals had significant reduction in the presence of inflammatory cells in nasal washes [66].

While ferret influenza mimics well the disease in humans, there are differences in drug pharmacokinetics in ferrets and humans. When testing oral compounds in animal models, differences in bioavailability among different species must be taken into consideration. As an example, oseltamivir phosphate – the ester prodrug of the active molecule oseltamivir carboxylate – is >75% orally bioavailable in humans [149], but only 30% bioavailable in mice and 11% in ferrets [150]; thus, dosages must be altered accordingly when assessing the activity of an oral compound in animal models. Similarly, different pharmacokinetics and toxicities can be observed in different species. In 1965, Cochran *et al*. found that the M2 ion channel inhibitor amantadine, when administered in doses tolerable in mice and men, caused lethal seizures in ferrets. At lower doses, experimental ferrets tolerated the drug, but those infected with the PR8 strain and treated either orally or subcutaneously with amantadine actually showed more viral lung pathology than infected animals given saline placebo [151]. Squires *et al*. later confirmed this report, showing that amantadine treatment had no ameliorating effect on either fever or clinical symptoms of sneezing, cough, and rhinorrhea [152]. In another study, Fenton *et al*. demonstrated similar toxicity and lack of efficacy in ferrets treated with oral amantadine; however, when treated with a lower dose of aerosolized amantadine, delivered by inhalation, ferrets infected with two different influenza A/H3N2 strains showed a reduction in nasal virus shedding and fever, without toxic effects [153]. Thus, the systemic toxicity of the amantadine limited the use of the ferret model to study its antiviral effects; when delivered locally, by inhalation, the drug worked as expected. Herlocher *et al*. later demonstrated that amantadine treatment (albeit at a lower dose than prior studies) also had no effect on development and duration of fever and or nasal virus shedding, but within five to six days of infection, the challenge virus developed amantadine resistance mutations in the M2 ion channel protein in four of nine amantadine-treated ferrets [154].

Early experiments by Francis and Magill demonstrated that ferrets inoculated with human influenza strains developed active immunity to re-infection, and that the serum of inoculated animals contained neutralizing antibodies, as evidenced by the capacity of the serum to confer passive protection to mice against infection with homologous strains [113]. Since then, many vaccine candidates have been tested in ferrets, including those against high pathogenicity avian influenza and the 2009 swine-origin pandemic viruses. Forrest *et al*. studied several inactivated whole-virus vaccine candidates against several clades of H5N1 avian influenza strains, including a multi-clade vaccine against four different H5N1 clades. These experiments found the vaccine candidates, when given in two doses, could protect against lethal virus challenge, prevent symptomatology (fever, lethargy, weight loss, and neurological signs), and reduced viral shedding from the upper respiratory tract [155].

#### 2.2.3. Transmission of influenza viruses in ferrets

In the 1930s, experiments demonstrated that influenza virus-naïve, asymptomatic ferrets co-caged with infected ferrets would subsequently develop the same disease; that after influenza virus infection, recovered ferrets were immune to experimental re-challenge with the epidemic strain; and that, even in the absence of experimental infection, ferrets occasionally displayed an influenza-like illness, after which they became immune to subsequent virus inoculation [113,119]. Since then, the ferret has been used as a model to study mammalian transmission of influenza virus and to test vaccines and drugs to prevent transmission and disease.

Because ferrets efficiently transmit human (unadapted) influenza strains, the model can be used to study viral and host factors that enhance mammalian transmission of human viruses, such as occur in periodic influenza pandemics. The influenza pandemics of 1957 and 1968 were caused by avian– human reassortant influenza viruses; however, the relative contribution of human internal protein genes or other molecular changes to the efficient transmission of influenza viruses among humans remains poorly understood. As in humans, most high pathogenicity avian influenza strains transmit poorly among ferrets. Maines *et al*. used the ferret model to assess whether reassortants between H5N1 avian and H3N2 human isolates might confer improved transmissibility among mammals. In this model, an H3N2 reassortant virus with avian virus internal protein genes exhibited efficient replication but inefficient transmission, whereas H5N1 reassortant viruses with four or six human virus internal protein genes exhibited reduced replication and no transmission. These results highlight the complexity of the genetic basis of influenza virus transmissibility and suggest that currently circulating avian H5N1 viruses may require further adaptation to acquire the enhanced mammalian transmissibility essential for pandemic spread [156].

The transmissibility of other avian influenza strains – those of the H7 [135] and H9 [136,157] subtypes – have been tested in the ferret model to assess their pandemic potential. Belser and colleagues compared H7 viruses of the Eurasian and North American lineages, assessing their receptor-binding preference by glycan array and then evaluating their transmissibility by direct contact in the ferret model. They found that two highly pathogenic avian viruses of the Eurasian lineage, both of which express H7 hemagglutinins with avian receptor-binding preference, demonstrated different transmissibility among ferrets; the first, NL/219, did not transmit between any of the three ferret pairs tested, while the second, NL/230, transmitted between two of three ferret pairs. In comparison, two North American lineage H7 viruses, NY/107 and Ck/Conn, both exhibited enhanced human receptor-binding preference and decreased binding to avian-type receptors, with NY/107 showing the most significant decrease in avian receptor preference. However, Ck/Conn transmitted poorly, between only one of three ferret pairs, while NY/107 transmitted very efficiently, between three of three ferret pairs [135]. These studies highlight the complex role of hemagglutinin receptor-binding preference in the mammalian transmissibility of influenza viruses. An avian receptor-binding preference, as measured by glycan-array technology, does not preclude reasonably efficient mammalian transmissibility of influenza viruses, nor is enhanced human receptor-binding preference alone sufficient for the efficient transmission of avian strains.

In ferret transmission studies with H9-subtype influenza viruses, Wan *et al*. assessed the mammalian transmissibility of wild-type H9N2 viruses. Though two of the five isolates transmitted between ferrets by direct contact, no aerosol transmission was observed. The authors found that a leucine residue at amino acid position 226 in the hemagglutinin receptor-binding site, responsible for human virus-like receptor specificity, was also important for ferret transmission [136]. The same group also studied a human-avian reassortant virus – expressing the surface proteins of an avian H9N2 virus in a human H3N2 backbone – which initially could not transmit via respiratory droplets among ferrets. However, after 10 serial passages of this reassortant virus through ferrets, the adapted virus acquired the ability transmit efficiently among ferrets in separate but adjacent cages. Sequence analysis of the passage-10 virus revealed only five amino acid changes relative to the passage-0 reassortant; experiments to identify the minimal changes necessary for transmission by respiratory droplet implicated three key changes in the HA and NA proteins [157]. These studies show that aerosol transmission is not exclusive to H1, H2, and H3 influenza subtypes, and they suggest that reassortants between human H3N2 and avian H9N2 viruses require little adaptation for efficient mammalian transmission by respiratory droplets.

More recently, the ferret model has been used to assess the transmissibility of the novel H1N1 swine-origin virus that began circulating among humans in 2009. Munster *et al*. [124] compared the transmissibility of the pandemic strain to a seasonal influenza H1N1 strain from 2007, and found that both viruses could transmit via respiratory droplets from an infected ferret to a naïve ferret housed near, but not in contact with, the infected animal. In this study, four pairs of ferrets were tested with each virus. Among the pairs inoculated with the pandemic H1N1 strain, virus was isolated from the nasopharynges of all four exposed animals. However, in those inoculated with the seasonal A/H1N1 virus, live virus could be isolated only from three of the four contact ferrets; in the fourth animal, viral nucleic acids were detected by reverse transcriptase-polymerase chain reaction (RT-PCR) alone. Although these data clearly showed that the pandemic H1N1 virus transmits efficiently, due to the small number of ferrets tested, it is unclear whether the pandemic virus may actually transmit better than the older seasonal strain.

Similarly, the ferret model has been used to compare the transmissibility of antiviral-resistant influenza A viruses. Yen *et al*. [158] compared the transmissibility of a wild-type H3N2 subtype influenza virus to that of two similar recombinant viruses, generated by reverse genetics, that each encoded a known oseltamivir resistance mutation in the neuraminidase (NA) gene. They found that one mutant virus, with a glutamic acid-to-valine substitution at position 119 of the viral NA (NA-E119V) transmitted similarly to the wild-type virus, but that the other mutant virus, with an arginine-to-lysine substitution at position 292 (NA-R292K) demonstrated poorer transmission efficiency. However, these experiments were performed with only one set of three ferrets for each virus, with one animal being the inoculated donor and the other two ferrets the naïve contact animals in the same cage; thus, two transmission events per virus, from a single donor animal, were assessed. While these experiments offer an important estimate of the public health threat posed by the resistant strains, the small number of animals used makes the effect of chance on the results difficult to assess.

These results highlight a drawback of the ferret model; namely, that the cost, size, and husbandry requirements of ferrets [142] make performing experiments with large numbers of animals difficult, and thus it can be prohibitively expensive to tease out small but statistically significant differences in transmissibility among different strains.

### 2.3. Guinea Pig (Cavia porcellus)

Outbred Hartley strain guinea pigs are used most commonly for influenza virus research. Although colonies of inbred strain 13 and strain 2 guinea pigs are still maintained, these animals are not commercially available. The strengths of the guinea pig model lie in the natural susceptibility of these animals to human influenza virus isolates, the efficiency with which human strains transmit among guinea pigs and the relative ease of obtaining, housing and working with these animals. The main drawback of the guinea pig model for influenza research is the lack of disease signs exhibited by infected animals.

#### 2.3.1. Susceptibility of guinea pigs to human and other influenza viruses

Like ferrets, guinea pigs are highly susceptible to infection with human influenza viruses, including seasonal strains of the H3N2 [112,159–163] and H1N1 [121,164] subtypes; 2009 pandemic strains [165]; the 1918 pandemic strain [164]; and highly pathogenic H5N1 viruses [164,166–168]. When administered intranasally, the 50% infectious dose of the seasonal strain A/Panama/2007/1999 (H3N2) was in the range of 5–66 PFU [112,167,169]. Influenza viral growth in guinea pigs occurs predominantly in the upper respiratory tract: following intranasal infection with 10^3^ PFU of A/Panama/2007/1999 (H3N2) virus, 10^7^ PFU/ml was obtained in nasal washings [112,165] on day 2 post-infection. The infection was cleared from the nasal passages by day 8. Growth in the lungs of guinea pigs was more moderate and shorter lived, with 10^5^ PFU/g isolated at three days and no virus detected at five days post-infection [112]. Guinea pigs can also be productively infected with avian and swine influenza isolates [164,165], although the titers reached by these viruses in nasal passages were generally lower than those seen with human strains.

In contrast to the situation in mice, the strain of guinea pig used for experimental influenza virus infections does not appear to alter the outcome. A/Panama/2007/1999 (H3N2) virus exhibited similar growth and transmission phenotypes in inbred strain 13 [121] and strain 2 [170] guinea pigs as in outbred Hartley guinea pigs.

#### 2.3.2. Signs of disease in guinea pigs

Despite the high viral titers reached in the respiratory tract, influenza viruses do not cause severe overt disease in guinea pigs. Their resistance to disease is demonstrated most strikingly with H5N1 strains that are highly pathogenic in humans and lethal to mice and ferrets at relatively low doses: guinea pigs infected with 10^6^ EID_50_ of A/Viet Nam/1203/2004 (H5N1) virus exhibited only mild listlessness [166], while those inoculated with the same dose of A/Thailand/16/2004 (H5N1) experienced a maximum of 7.3% body weight loss [164]. Consistent with this relative lack of disease, highly pathogenic H5N1 viruses do not appear to spread systemically in guinea pigs: virus was not detected in the spleen, kidney, colon or brain of intranasally inoculated animals [168], and productive infection did not result from intragastric inoculation [166]. Infection of guinea pigs with epidemic human strains or 2009 pandemic H1N1 viruses does not lead to fever, weight loss, lethargy, coughing or sneezing; increased production of mucus in the nasal passages is, however, apparent starting approximately four days after infection.

Despite this relative lack of discernible symptoms, examination of respiratory tissues derived from infected guinea pigs reveals significant histopathological changes. Mild to severe bronchointerstitial pneumonia characterized by the infiltration of immune cells and destruction of ciliated epitheilia have been reported following infection with a number of different influenza viruses [163,164,166,171]. Among the viruses they tested, Van Hoeven *et al*. reported that the severity of lung pathology was greater for the 1918 pandemic strain than A/Thailand/16/2004 (H5N1) virus, which in turn caused more severe lesions than A/duck/Alberta/35/1976 (H1N1) and A/Texas/36/1991 (H1N1) viruses [164]. Examination of the upper airways, the main site of viral replication, in infected animals revealed rhinitis characterized by heavy nasal mucus secretion [163].

#### 2.3.3. Transmission of influenza viruses among guinea pigs

Human influenza viruses transmit efficiently from guinea pig-to-guinea pig. Rapid transmission is seen when infected and naïve guinea pigs are housed in the same cage together (*i.e.,* direct contact transmission) and also when animals are placed in separate but adjacent cages (referred to herein as aerosol transmission, mediated by either large respiratory droplets or small, airborne, droplets) [112]. Although it appears to be less efficient than close-range spread, evidence for transmission of the virus A/Panama/2007/1999 (H3N2) by the airborne route has been obtained in the guinea pig model: Mubareka *et al*. reported transmission over a distance of three feet, and also in an upward direction [121].

Viral strain and subtype specific differences in transmission have been observed in guinea pigs. In general, seasonal H1N1 subtype viruses have been found to transmit less efficiently than epidemic strains of the H3N2 subtype [121,170]. Both 2009 pandemic H1N1 isolates tested to date transmitted very efficiently, however [165]. The human isolate A/Viet Nam/1203/2004 (H5N1) spread to three of four exposed guinea pigs when infected and naïve animals were co-caged [167], but did not transmit by the aerosol route [172]. Among six avian isolates of highly pathogenic H5N1 influenza examined by Gao *et al*., two were seen to spread from guinea pig-to-guinea pig by the direct contact route [168]. Finally, swine influenza isolates of the H1 and H3 subtypes transmitted with 25% efficiency by the aerosol route [165,172], while the low pathogenic avian strains A/duck/Alberta/35/1976 (H1N1) and A/duck/Ukraine/1963 (H3N8) did not transmit [170].

Since signs of disease in guinea pigs are not easily monitored, this model is not commonly used for the evaluation of antiviral drugs or vaccines in terms of the reduction in influenza symptoms they might achieve. Viral titers in infected animals and transmission to contacts, by contrast, can be quantified through very simple procedures that do not require the harvesting of tissues. Namely, the collection of nasal washings over a time course of exposure and/or serological analysis performed two weeks or more after the exposure are used to monitor transmission. In this way, the guinea pig model can be used to assess the efficacy of various interventions in decreasing viral load and limiting influenza virus transmission [160,164,165]. Similarly, the guinea pig is a convenient model in which to examine the potential for drug resistant strains of influenza to spread. By performing relatively simple transmission experiments using either the wild type A/Panama/2007/1999 (H3N2) virus or recombinant strains in which oseltamivir resistance mutations had been introduced, we found that the mutant viruses examined transmitted with equal efficiency to the wild type by a contact route, but transmitted poorly or not at all by aerosol [169].

### 2.4. Cotton Rat (Sigmodon hispidus)

A well-established model for respiratory syncitial virus, the cotton rat has also been characterized as a model for human influenza viruses. As with ferrets and guinea pigs, one of the main advantages of the cotton rat model is that it can be used with human influenza A and B viruses with no prior adaptation. A second attraction is that inbred cotton rats are available. To our knowledge, no data on the transmission of influenza viruses among cotton rats has been published; instead, studies to date in this model have focused on lower respiratory tract disease. Following intranasal inoculation with a high viral dose, cotton rats develop a number of signs of disease which can be evaluated quantitatively, thereby allowing pathogenicity and its reduction through treatment to be assessed.

#### 2.4.1 Susceptibility to human and other influenza viruses

Cotton rats have been shown to be susceptible to the seasonal human strains, A/Wuhan/359/1995 (H3N2) and A/Malaya/302/1954 (H1N1); the laboratory adapted viruses PR8 and X31; and influenza B viruses B/Sichuan/379/99, B/HK/330/01, and B/HK/73 [173]. The 50% infectious dose of A/Wuhan/359/1995 (H3N2) virus was 10^2^ TCID_50_ [173]. Peak viral titers isolated from lung were reached at one day post-infection, were found to be dose-dependent, and in each case the infectious units per gram of tissue did not exceed the dose administered. Titers reached in nasal tissues, by contrast, were in the range of 10^6^–10^7^ TCID_50_/g, regardless of the inoculum dose, and were reached at three days post-infection [173]. Thus, the predominant site of viral replication in the cotton rat is the upper respiratory tract.

#### 2.4.2. Signs of disease in cotton rats

Following infection with 10^7^ TCID_50_ of A/Wuhan/359/1995 (H3N2) virus, cotton rats developed hypothermia on days 1, 2 and three post-infection, lost a maximum of 10% of their initial body weight, and experienced an increase in respiratory rate (tachypnea) of 90% [173,174]. The severity of tachypnea observed correlated with the inoculum dose administered.

Despite detection of high viral titers in the nose, and relatively lower titers in the lungs, histopathological analysis of influenza virus infected cotton rats revealed lesions predominantly in the lower respiratory tract [173]. Thus, following infection with 10^7^ TCID_50_ of A/Wuhan/359/1995 virus, few nasal lesions and no nasal inflammation were observed. In the larger airways of the lung, the columnar epithelium was seen to slough off already at day 2 post-infection, while interstitial pneumonia and alveolitis were apparent at day four post-infection [173]. The destruction of small airway epithelia was furthermore seen to correlate with the development of tachypnea [174].

The ability to monitor changes in breathing rate using whole body plethysmography provides a convenient and quantitative measure of disease in influenza-infected cotton rats. Using this assay, and evaluations of lung pathology, the cotton rat model has been used to test the efficacy of anti-inflammatory and anti-viral treatments. Administration of the neuraminidase inhibitors, zanamivir or oseltamivir, in two different post-infection treatment regimens did not significantly reduce tachypnea or pathology scores in animals infected with 10^7^ TCID_50_ of A/Wuhan/359/1995 virus [174,175]. It should be noted, however, that the effective dose of these drugs administered to each animal is unclear since their bioavailability in the cotton rat has not been determined. The combination of zanamivir or oseltamivir with convalescent serum or an anti-inflammatory compound, triamcinolone acetonide, applied intranasally was, however, found to significantly reduce lung lesions [175]. The cotton rat model has furthermore been used to demonstrate that pre-existing immunity to an H1N1 subtype influenza virus reduced tachypnea and bronchiolar epithelial cell damage upon challenge with A/Wuhan/359/1995 (H3N2) virus [176,177].

### 2.5. Syrian (Golden) Hamsters (Mesocricetus auratus)

In the 1940s, experiments showed that hamsters inoculated with influenza viruses demonstrated no clinical signs of disease, yet they mounted a specific antibody response to the infection [178]. In the 1960s through 1980s, the hamster model was employed primarily in studies of vaccine efficacy; however, the model has been used less frequently in recent years. Like cotton rats and guinea pigs, hamsters are small and relatively inexpensive to maintain, and they are susceptible to infection with human influenza viruses without prior adaptation to the species. Influenza virus infection in hamsters is a primarily upper respiratory tract infection, and virus is easily recoverable from nasal washes, allowing serial observations of the course of infection to be made on single animals. However, like guinea pigs, influenza disease in hamsters is clinically unapparent, and thus symptoms cannot be used to assess the progression of infection or the amelioration of disease with antivirals or vaccines.

#### 2.5.1. Susceptibility of hamsters to human and other influenza viruses

Like ferrets, guinea pigs and cotton rats, hamsters are naturally susceptible to unadapted human influenza virus isolates, including influenza A subtypes H1N1 [179–181], H2N2 [179,180,182], and H3N2 [179–181,183–185], and influenza B viruses [180,186].

#### 2.5.2. Signs of disease in hamsters

In early studies [181,185], hamsters inoculated with influenza viruses did not show signs or symptoms of infection, but influenza virus-specific antibodies could be serologically demonstrated after infection. Like humans, ferrets, and guinea pigs, influenza infection in hamsters is a primarily upper respiratory tract infection, and nasal washes yield high titers of influenza virus, providing a relatively non-invasive method for tracking the progress of influenza virus infection over time in a single animal, without requiring the euthanasia of animals at daily time points.

In hamsters inoculated by aerosol with H3N2 subtype influenza strains, virus could be recovered from lung and nasal tissues, with highest titers seen on day 2 to 3 post-inoculation and decreasing sharply on subsequent days, with no virus detectable seven days post-inoculation. This pattern is similar to that seen in guinea pigs. Anti-hemagglutinin antibodies could be detected in the serum of hamsters five days post-inoculation, with highest titers two weeks post-inoculation [185].

In evaluating the hamster model for the study of vaccines, Potter and Jennings found that hamsters were susceptible to intranasal inoculation with A/England/42/72 (H3N2) virus, and virus was recoverable from both lung homogenates and nasal washes taken post-inoculation; nasal wash titers were approximately ten-fold higher than lung titers at each time point. All infected hamsters also mounted detectable serum anti-hemagglutinin antibody responses, and infected animals were protected from re-infection with homologous, but not heterologous, virus challenge. However, infected animals displayed no significant increase in rectal temperature, no antibody response could be detected in nasal washes, and no lung pathology was seen upon necropsy [181]. Similarly, in hamsters infected by aerosol with an influenza B strain (B/Hong Kong/8/73), virus could be isolated from both nasal and lung tissues, but neither clinical signs of infection nor lung lesions were observed [186].

Hamsters have been utilized in vaccine studies, especially for the study of live-attenuated influenza virus vaccines. Live viruses can be attenuated by passaging them at decreasing temperatures, thus adapting them to grow optimally at lower temperatures and creating a “cold-adapted” or “temperature-sensitive” phenotype. A virus adapted to grow at 33 °C, when inoculated into the nose of a vaccinee, will replicate well in the nasopharynx and stimulate an immune response, but lower respiratory tract replication at 37 °C will be inhibited. Like humans, the core body temperature of the hamster is 37 °C, so they are an ideal species to study the efficacy of cold-adapted virus vaccines. In 1971, Mills *et al*. demonstrated that an H2N2 virus, cold-adapted *in vitro*, also displays an attenuated phenotype in hamsters. Whereas the wild-type virus replicated to high lung titers within one day of intranasal inoculation, cold-adapted viruses achieved peak lung titers that were 10 to 1000-fold lower and one to two days later than the parental virus [182]. Abou Donia *et al*. later confirmed that temperature-sensitive influenza virus mutants showed reduced growth in the lungs, but not the nasal turbinates, of hamsters, similar to the phenotype of these viruses in man [183].

In 1974, Jennings *et al.* studied the hemagglutination-inhibiting and neuraminidase-inhibiting antibody response of hamsters to inactivated influenza virus vaccines. They found that the antibody response was enhanced not only by prior infection with live, heterosubtypic influenza A viruses but also by prior immunization with inactivated, adjuvanted, heterosubtypic influenza A virus vaccines. Thus, hamsters pre-infected with a live virus required a lower dose of heterosubtypic vaccine to stimulate the equivalent antibody response as an uninfected, vaccinated animal. However, in hamsters, the priming effect was lost when live-virus pre-infection preceded inactivated-virus vaccination by 20 weeks or more, although antibodies to the pre-infecting virus could still be detected out to 32 weeks post-infection. In contrast, no priming effect was seen with influenza B viruses, although infection with these viruses did occur, as demonstrated by serum hemagglutination inhibition after infection [180].

#### 2.5.3. Transmission of influenza viruses in hamsters

In 1982, Ali *et al*. [179] investigated the transmissibility of several influenza A viruses among hamsters. In a contact transmission model, donor hamsters were inoculated with virus and were then placed in a cage with naïve recipient hamsters at one hour post-inoculation. Transmission was defined by seroconversion in the exposed recipient hamsters, as a four-fold rise in anti-hemagglutinin antibody titer at 14–21 days post-inoculation. They found that viruses more virulent in man tended to achieve higher nasopharyngeal titers and to transmit more efficiently to naïve hamsters (Table 2). However, the relationship between maximal nasal wash titer and transmissibility was not absolute; the viruses A/Finland/74 (H3N2) and A/USSR/77 (H1N1) achieved similar nasal wash titers, but the transmissibility was vastly different, with 100% transmission (11 of 11 hamsters) by the H3N2 virus and 0% (0 of 11 hamsters) by the H1N1 strain.

Although the data was not shown, the authors noted that aerosol transmission – in which the infected and naïve hamsters were not allowed to touch, but were instead separated by a space of one inch – did not proceed among hamsters with any of the viruses tested.

### 2.6. Nonhuman Primates: Rhesus macaque (Macaca mulatta), Pig-tailed macaque (Macaca nemestrina), and Cynomolgus macaque (Macaca fascicularis)

Because of the genetic and physiological similarities between human and nonhuman primates, macaques are thought to more closely model the human response to influenza virus infection than do more distantly related mammalian species like mice and ferrets. Thus, nonhuman primates have been used to study highly pathogenic influenza virus infections, such as those caused by avian H5N1 and the 1918 pandemic viruses, in which the host’s cytokine response to infection is thought to play a role in disease pathogenesis, as well as to study the efficacy of antiviral medications and vaccinations against these pathogenic strains. However, their high cost, complex husbandry requirements, relatively low availability compared to species more easily bred in captivity, and ethical issues with their use make nonhuman primates less accessible for routine studies of influenza virus pathogenicity and transmission than the previously discussed animal models. Indeed, to our knowledge, the transmissibility of influenza viruses among macaques has not been assessed.

#### 2.6.1. Susceptibility of nonhuman primates to human and other influenza viruses

Nonhuman primates are susceptible to infection with a number of unadapted human influenza A isolates, including viruses of the H1N1 (including pre-2009 seasonal [39,187–192] and 2009 swine-origin pandemic [39] strains, as well as the reconstructed 1918 pandemic virus [191,193]), H3N2 [194], and H5N1 [188,193,195–199] subtypes.

#### 2.6.2. Signs of disease in nonhuman primates

Within a decade of the first isolation of influenza virus from humans into ferrets, experimental inoculation of nonhuman primates had been performed. In 1946, Saslaw *et al.* intranasally inoculated rhesus macaques with the PR8 virus, a human strain passaged through mice. No clinical signs of infection, including fever, anorexia, debility, or the respiratory distress were observed; however, infected monkeys demonstrated leukopenia, primarily manifesting as a decrease in neutrophils, and developed neutralizing antibodies to the inoculating strain between eight and 10 days post-infection. However, in two monkeys inoculated by instillation of virus via syringe directly into the trachea, signs and symptoms consistent with influenza were observed, including listlessness and lethargy, facial flushing, and conjunctival injection. Symptoms persisted for two days, after which the animals returned to baseline. In these animals too, neutropenia with reciprocal lymphocytosis was seen [192].

A more pronounced clinical syndrome is observed in nonhuman primates infected with highly pathogenic human viruses, including avian influenza H5N1 strains. Cynomolgus macaques inoculated with an HPAI H5N1 virus developed fever within two days of infection. One of four monkeys developed clinical signs consistent with the acute respiratory disease syndrome (ARDS), including tachypnea, cough, lethargy, anorexia, and peripheral cyanosis. On necropsy of monkeys euthanized four or seven days after infection, high viral titers were isolated from lung tissue, which demonstrated necrotizing bronchointerstitial pneumonia, including extensive loss of alveolar and bronchiolar epithelium with alveolar exudates comprising edema, fibrin, cell debris, and peripheral blood cells. This pathology is similar to that seen in primary influenza pneumonia in humans [199].

Baskin *et al*. assessed lung pathology in cynomolgus macaques infected with a HPAI H5N1 virus, a seasonal human influenza strain A/Texas/36/1991 (H1N1) (Tx91), and a recombinant virus expressing the six internal genes of Tx91 with the hemagglutinin and neuraminidase from the reconstructed 1918 pandemic influenza virus. Macaques infected with the H5N1 virus demonstrated a more severe clinical syndrome than those infected with either Tx91 or the Tx91-1918 reassortant; clinical obervations included anorexia, depression, coughing, diarrhea, and thrombocytopenia in H5N1-infected animals. Both the H5N1 and Tx91-1918 recombinant viruses produced severe pathology, a multi-lobar bronchopneumonia with consolidation and edema on gross inspection and bronchiolitis and alveolitis on microscopy, though pathology was worse in the H5N1 virus infected animals [188].

A subsequent study by Cilloniz *et al*. compared lung pathology in cynomolgus macaques infected with an HPAI H5N1 virus to those infected with the 1918 pandemic virus. They found that animals infected with the 1918 virus demonstrated more severe lung pathology within the first 24 hours of infection, with severe peribronchiolar alveolitis, edema, and hemorrage. By 48 hours post-inoculation (hpi), similar lung pathology was seen with both viruses. Lung titers were higher at 12 hpi in 1918-infected macaques, but titers equalized for both viruses by 24 hpi. Thus, although virus titers were similar at the 24-hour time point, the 1918 virus had already caused greater tissue damage, which continued to worsen [193].

In a recent study comparing the new swine-origin pandemic strain, A/California/04/2009 (H1N1), to an older seasonal H1N1 virus, Itoh *et al*. found that cynomolgus macaques infected with the latter manifested a greater febrile response to infection, had higher virus titers in both the upper and lower respiratory tracts, and demonstrated more severe lung lesions on pathological examination, compared to macaques infected with the seasonal virus [39].

Despite the genetic and physiologic similarities between human and nonhuman primates, there are likely subtle differences in influenza virus infection in these species. Using virus histochemistry, Van Riel *et al*. found that, in both humans and macaques, avian viruses attach more strongly to cells in the lower respiratory tract; however, in human alveoli, avian viruses tend to attach to Type II (surfactant-producing) pneumocytes, while in macaques, viral binding was predominantly to Type I pneumocytes [146].

Antivirals and vaccines against influenza virus have also been tested in the nonhuman primate model. Recently, Stittelaar *et al*. evaluated the therapeutic efficacy of intravenous zanamavir in H5N1 avian influenza virus infection. In the United States, zanamavir is currently licensed for use as an inhaled preparation; however, this dosing method is impractical for patients critically ill with avian influenza. In addition, zanamivir is the only currently available neuraminidase inhibitor effective against H1N1 viruses with the NA-H275Y mutation. Stittelaar *et al*. found a dose-dependent antiviral efficacy in reducing viral titers in the lungs of infected macaques who were treated with IV zanamivir either prophylactically (administered starting 12 hours prior to infection) or therapeutically (starting four hpi), although there was significant variation among lung titers from animals in each group. Additionally, fewer zanamivir-treated animals developed lung lesions than did those in the placebo group, and, in treated animals that developed pneumonia, the pathology was generally less severe [200].

Because of the genetic and physiologic similarities between humans and nonhuman primates, several studies [187,188,191,193] have explored gene expression during influenza virus infection in macaques. Such a study in rhesus macaques found that oseltamivir prophylaxis, prior to infection with a seasonal H1N1 influenza isolate, significantly reduced virus titers in the trachea and also increased mRNA levels of the interferon-stimulated gene *MxA*; from this data, the authors hypothesized that, in primates, influenza virus protein expression actively suppresses expression of this antiviral gene [189].

Nonhuman primates have also been used to assess the immunogenicity and efficacy of vaccines against influenza virus infection. These experiments have shown efficacy – measured either by the induction of protective antibody and cellular immune responses or the reduction of disease in vaccinated, virus-challenged animals – of a number of novel vaccine candidates. Some of these novel strategies include modifications of existing vaccines, such as a cold-adapted live-attenuated influenza virus vaccine (LAIV) directed against highly pathogenic avian influenza viruses [195]. A novel LAIV rendered replication-deficient not by cold-adaptation, but by the deletion of a viral gene that antagonizes the protective interferon response, has also been tested [201]. Other novel strategies include intradermal or intramuscular electroporation of plasmid DNA-encoded antigens [197] or intramuscular administration of replication-deficient vaccinia virus as a vector to deliver influenza virus genes [196].

## 3. Conclusions

Several animal species have been used in influenza virus research, each with particular advantages and disadvantages. Symptoms of influenza virus infection in humans are most closely mimicked by the ferret, in which influenza virus disease is manifested by fever, nasal discharge, lethargy, weakness, anorexia, and sneezing. Depending on the virus strain, macaques can also display a human-like symptomatology, and, like humans, infection with highly pathogenic avian influenza viruses can induce ARDS and multi-organ system dysfunction in primate models. Mice and cotton rats, while less overtly symptomatic, can display hypothermia and weight loss, while guinea pigs and hamsters show no overt clinical signs of influenza virus infection. Thus, the use of antivirals to prevent symptoms cannot be studied adequately in the rodent models.

Ferrets, guinea pigs, cotton rats, hamsters, and nonhuman primates are all susceptible to infection with human influenza virus strains, without the need for prior adaptation to the species. Mice, however, are resistant to infection with most primary human virus isolates; thus, they are less useful when studying non-adapted strains. Important exceptions include the 1918 H1N1 pandemic strain, highly pathogenic avian influenza viruses, some low pathogenic avian influenza viruses, and the 2009 H1N1 pandemic strain, which have been demonstrated to be infectious in mice without prior passaging.

Efficient transmission of influenza virus has been shown only in ferrets, guinea pigs, and, to a lesser extent, hamsters. While nonhuman primates would be expected to transmit human strains like humans, the large numbers of animals required for these experiments would be cost-prohibitive. Thus, for studies on mammalian transmission of influenza virus and interventions aimed at preventing spread, most of the models discussed in this review, with the exception of ferrets and guinea pigs, would be unsuitable.

In terms of size, cost, and husbandry requirements, the smaller rodents – mice, cotton rats, hamsters, and guinea pigs – are readily accessible to most researchers, and statistically robust data may be obtained with large numbers of animals. Ferrets and nonhuman primates are more expensive, require larger caging and facilities, and have greater husbandry demands than the smaller models, thus limiting their use in some research settings.

## 4. Future Perspectives

The heightened interest of the public and funding bodies in influenza in recent years has led to the expansion of the field and, correspondingly, a greater need for well characterized animal models of disease and transmission. While, as discussed above, significant progress has been achieved in this regard, challenges remain. One important topic which has been difficult to fully address in either humans or animal models is the relative contributions of small droplet aerosols, larger respiratory droplets, and contact with contaminated surfaces to influenza virus transmission. Technological hurdles related to the isolation and quantification of viable influenza viruses from dilute aerosols have hampered progress on this issue. If these can be overcome, the insight gained will be of great value in informing public health responses to influenza virus outbreaks.

## Figures and Tables

**Table 1.** Susceptibility of standard inbred mice to influenza viruses following intranasal inoculation.

Viral Strain *	50% infectious dose (LD_50_) **	50% lethal dose (LD_50_) **	**Mouse Strain**	**Reference**
PR8 (H1N1)		10^2^ PFU	BALB/C	[40]
WSN (H1N1)		10^2^ – 10^3.3^ PFU	BALB/C	[40,74,75]
X31 (H3N2)	10^0.7^ EID_50_	>10^5.2^ EID_50_, 10^5.84^ PFU	BALB/C	[40,48]
1918 pandemic strain (H1N1)	10^0.75^ PFU	10^3.25^ to 10^3.5^ PFU	BALB/C	[36,57]
A/New Caledonia/20/1999 (H1N1)	∼10^2.7^ PFU		C57BL/6	[37]
A/Texas/36/1991 (H1N1)		>10^6^ PFU	BALB/C	[36]
A/Kawasaki/UTK-4/09 (H1N1)		>10^6.6^ PFU	BALB/C	[39]
A/Netherlands/607/2009 (pH1N1)		<10^4.7^ PFU	C57BL/6	[73]
A/California/04/2009 (pH1N1)	10^1.5^	10^4.7^ to >10^6^ PFU	BALB/C	[39,57,73]
A/Viet Nam/1203/2004 (H5N1)	10^1.5^ PFU, 10^2.2^ EID_50_	10^1.3^ PFU, 10^1.8^ EID_50_	BALB/C	[57,76]
A/Hong Kong/483/1997 (H5N1)	10^2.2^ EID_50_	10^1.6^ to 10^2.4^ EID_50_	BALB/C	[48,76]
A/chicken/BC/CN-7/04 (H7N3)		10^2.4^ TCID_50_	BALB/C	[55]
A/Netherlands**/**219/03 (H7N7)	10^0.76^ EID_50_	10^2.5^ EID_50_, 10^0.8^ TCID_50_	BALB/C	[53,55]
A/turkey/VA/4529/02 (H7N2)	10^1.76^ EID_50_	>10^7^ TCID_50_	BALB/C	[53]
A/Red Knot/NJ/1523470/06 (H7N3)	10^1.5^ PFU	>10^4.8^ PFU	BALB/C	[56]
A/Ruddy Turnstone/DE/650645/02 (H2N9)	10^2.4^ PFU	>10^5.4^ PFU	BALB/C	[56]

* pH1N1 indicates H1N1 subtype viruses of the 2009 pandemic

** PFU = plaque forming units; EID_50_ = 50% egg infectious dose; TCID_50_ = 50% tissue culture infectious dose

**Table 2.** Transmission of human influenza A viruses in hamsters. (Adapted from [179].)

**Virus**	**Maximum nasal wash titer [log_10_ 50% egg-bit infectivity dose (EBID_50_)/mL]**	**Transmission Efficiency [# transmissions/ # exposed, (%)]**
*Virulent in humans*:		
A/Finland/74 (H3N2)	6.2	5/5 (100%)
A/Victoria/75 (H3N2)	5.5	5/5, 6/6 (100%)
A/Texas/77 (H3N2)	5.9	5/5, 4/5 (90%)

*Attenuated in humans*:		
A/PR/8/34 (H1N1)	4.6	3/5 (60%)
A/Okuda/57 (H2N2)	4.3	0/5, 0/5 (0%)
A/HK/l19/77 (H1N1)	5.3	2/5 (40%)
A/U.S.S.R./77 (H1N1)	5.3	0/6, 0/5 (0%)

## References

[1] Treanor JJ, Mandell GL, Bennett JE, Dolin R (2010). Influenza Viruses, Including Avian Influenza and Swine Influenza. Mandell, Douglas, and Bennett's Principles and Practices of Infectious Diseases.

[2] Lapinsky SE (2010). Epidemic viral pneumonia. Curr Opin Infect Dis.

[3] Murata Y, Walsh EE, Falsey AR (2007). Pulmonary complications of interpandemic influenza A in hospitalized adults. J Infect Dis.

[4] Carrat F, Vergu E, Ferguson NM, Lemaitre M, Cauchemez S, Leach S, Valleron AJ (2008). Time lines of infection and disease in human influenza: a review of volunteer challenge studies. Am J Epidemiol.

[5] Bjornson AB, Mellencamp MA, Schiff GM (1991). Complement is activated in the upper respiratory tract during influenza virus infection. Am Rev Respir Dis.

[6] Couch RB, Douglas RG, Fedson DS, Kasel JA (1971). Correlated studies of a recombinant influenza-virus vaccine. 3. Protection against experimental influenza in man. J Infect Dis.

[7] Whicher JT, Chambers RE, Higginson J, Nashef L, Higgins PG (1985). Acute phase response of serum amyloid A protein and C reactive protein to the common cold and influenza. J Clin Pathol.

[8] Cauchemez S, Donnelly CA, Reed C, Ghani AC, Fraser C, Kent CK, Finelli L, Ferguson NM (2009). Household transmission of 2009 pandemic influenza A (H1N1) virus in the United States. N Engl J Med.

[9] France AM, Jackson M, Schrag S, Lynch M, Zimmerman C, Biggerstaff M, Hadler J (2010). Household transmission of 2009 influenza A (H1N1) virus after a school-based outbreak in New York City, April–May 2009. J Infect Dis.

[10] Morgan OW, Parks S, Shim T, Blevins PA, Lucas PM, Sanchez R, Walea N, Loustalot F, Duffy MR, Shim MJ (2010). Household transmission of pandemic (H1N1) 2009, San Antonio, Texas, USA, April–May 2009. Emerg Infect Dis.

[11] Fabian P, McDevitt JJ, DeHaan WH, Fung RO, Cowling BJ, Chan KH, Leung GM, Milton DK (2008). Influenza virus in human exhaled breath: an observational study. PLoS One.

[12] Stelzer-Braid S, Oliver BG, Blazey AJ, Argent E, Newsome TP, Rawlinson WD, Tovey ER (2009). Exhalation of respiratory viruses by breathing, coughing, and talking. J Med Virol.

[13] Frank AL, Taber LH, Wells CR, Wells JM, Glezen WP, Paredes A (1981). Patterns of shedding of myxoviruses and paramyxoviruses in children. J Infect Dis.

[14] Glezen WP (2006). Influenza control. N Engl J Med.

[15] Hall CB (1981). Nosocomial viral respiratory infections: perennial weeds on pediatric wards. Am J Med.

[16] Hall CB, Douglas RG, Geiman JM, Meagher MP (1979). Viral shedding patterns of children with influenza B infection. J Infect Dis.

[17] Sato M, Hosoya M, Kato K, Suzuki H (2005). Viral shedding in children with influenza virus infections treated with neuraminidase inhibitors. Pediatr Infect Dis J.

[18] Weinstock DM, Gubareva LV, Zuccotti G (2003). Prolonged shedding of multidrug-resistant influenza A virus in an immunocompromised patient. N Engl J Med.

[19] Buxton Bridges C, Kuehnert MJ, Hall CB (2003). Transmission of influenza: implications for control in health care settings. Clin Infect Dis.

[20] Alford RH, Kasel JA, Gerone PJ, Knight V (1966). Human influenza resulting from aerosol inhalation. Proc Soc Exp Biol Med.

[21] Brankston G, Gitterman L, Hirji Z, Lemieux C, Gardam M (2007). Transmission of influenza A in human beings. Lancet Infect Dis.

[22] Tellier R (2006). Review of aerosol transmission of influenza A virus. Emerg Infect Dis.

[23] Staeheli P, Grob R, Meier E, Sutcliffe JG, Haller O (1988). Influenza virus-susceptible mice carry Mx genes with a large deletion or a nonsense mutation. Mol Cell Biol.

[24] Haller O (1981). Inborn resistance of mice to orthomyxoviruses. Curr Top Microbiol Immunol.

[25] Haller O, Arnheiter H, Gresser I, Lindenmann J (1979). Genetically determined, interferon-dependent resistance to influenza virus in mice. J Exp Med.

[26] Staeheli P, Haller O, Boll W, Lindenmann J, Weissmann C (1986). Mx protein: constitutive expression in 3T3 cells transformed with cloned Mx cDNA confers selective resistance to influenza virus. Cell.

[27] Grimm D, Staeheli P, Hufbauer M, Koerner I, Martinez-Sobrido L, Solorzano A, Garcia-Sastre A, Haller O, Kochs G (2007). Replication fitness determines high virulence of influenza A virus in mice carrying functional Mx1 resistance gene. Proc Natl Acad Sci U S A.

[28] Tumpey TM, Szretter KJ, Van Hoeven N, Katz JM, Kochs G, Haller O, Garcia-Sastre A, Staeheli P (2007). The Mx1 gene protects mice against the pandemic 1918 and highly lethal human H5N1 influenza viruses. J Virol.

[29] Alberts R, Srivastava B, Wu H, Viegas N, Geffers R, Klawonn F, Novoselova N, do Valle TZ, Panthier JJ, Schughart K (2010). Gene expression changes in the host response between resistant and susceptible inbred mouse strains after influenza A infection. Microbes Infect.

[30] Boon AC, deBeauchamp J, Hollmann A, Luke J, Kotb M, Rowe S, Finkelstein D, Neale G, Lu L, Williams RW (2009). Host genetic variation affects resistance to infection with a highly pathogenic H5N1 influenza A virus in mice. J Virol.

[31] Srivastava B, Blazejewska P, Hessmann M, Bruder D, Geffers R, Mauel S, Gruber AD, Schughart K (2009). Host genetic background strongly influences the response to influenza a virus infections. PLoS One.

[32] Hale BG, Steel J, Manicassamy B, Medina RA, Ye J, Hickman D, Lowen AC, Perez DR, Garcia-Sastre A (2010). Mutations in the NS1 C-terminal tail do not enhance replication or virulence of the 2009 pandemic H1N1 influenza A virus. J Gen Virol.

[33] Boon AC, deBeauchamp J, Krauss S, Rubrum A, Webb AD, Webster RG, McElhaney J, Webby RJ (2010). Cross-reactive neutralizing antibodies directed against pandemic H1N1 2009 virus are protective in a highly sensitive DBA/2 mouse influenza model. J Virol.

[34] Hai R, Schmolke M, Varga ZT, Manicassamy B, Wang TT, Belser JA, Pearce MB, Garcia-Sastre A, Tumpey TM, Palese P (2010). PB1-F2 expression by the 2009 pandemic H1N1 influenza virus has minimal impact on virulence in animal models. J Virol.

[35] Solorzano A, Ye J, Perez DR (2010). Alternative live-attenuated influenza vaccines based on modifications in the polymerase genes protect against epidemic and pandemic flu. J Virol.

[36] Tumpey TM, Basler CF, Aguilar PV, Zeng H, Solorzano A, Swayne DE, Cox NJ, Katz JM, Taubenberger JK, Palese P (2005). Characterization of the reconstructed 1918 Spanish influenza pandemic virus. Science.

[37] Glaser L, Conenello G, Paulson J, Palese P (2007). Effective replication of human influenza viruses in mice lacking a major alpha2,6 sialyltransferase. Virus Res.

[38] Hoyle L, Gard S, Hallauer C, Meyer KF (1968). The Influenza Viruses.

[39] Itoh Y, Shinya K, Kiso M, Watanabe T, Sakoda Y, Hatta M, Muramoto Y, Tamura D, Sakai-Tagawa Y, Noda T (2009). *In vitro* and *in vivo* characterization of new swine-origin H1N1 influenza viruses. Nature.

[40] Quan FS, Steinhauer D, Huang C, Ross TM, Compans RW, Kang S-M (2008). A bivalent influenza VLP vaccine confers complete inhibition of virus replication in lungs. Vaccine.

[41] Friede M, Muller S, Briand JP, Plaue S, Fernandes I, Frisch B, Schuber F, Van Regenmortel MH (1994). Selective induction of protection against influenza virus infection in mice by a lipid-peptide conjugate delivered in liposomes. Vaccine.

[42] Mitchell JA, Green TD, Bright RA, Ross TM (2003). Induction of heterosubtypic immunity to influenza A virus using a DNA vaccine expressing hemagglutinin-C3d fusion proteins. Vaccine.

[43] Muller S, Plaue S, Samama JP, Valette M, Briand JP, Van Regenmortel MH (1990). Antigenic properties and protective capacity of a cyclic peptide corresponding to site A of influenza virus haemagglutinin. Vaccine.

[44] Saelens X, Vanlandschoot P, Martinet W, Maras M, Neirynck S, Contreras R, Fiers W, Jou WM (1999). Protection of mice against a lethal influenza virus challenge after immunization with yeast-derived secreted influenza virus hemagglutinin. Eur J Biochem.

[45] Simeckova-Rosenberg J, Yun Z, Wyde PR, Atassi MZ (1995). Protection of mice against lethal viral infection by synthetic peptides corresponding to B- and T-cell recognition sites of influenza A hemagglutinin. Vaccine.

[46] Stevenson PG, Hawke S, Bangham CR (1996). Protection against lethal influenza virus encephalitis by intranasally primed CD8+ memory T cells. J Immunol.

[47] Wraith DC, Vessey AE, Askonas BA (1987). Purified influenza virus nucleoprotein protects mice from lethal infection. J Gen Virol.

[48] Lu X, Tumpey TM, Morken T, Zaki SR, Cox NJ, Katz JM (1999). A mouse model for the evaluation of pathogenesis and immunity to influenza A (H5N1) viruses isolated from humans. J Virol.

[49] Hai R, Martinez-Sobrido L, Fraser KA, Ayllon J, Garcia-Sastre A, Palese P (2008). Influenza B virus NS1-truncated mutants: live-attenuated vaccine approach. J Virol.

[50] Gao P, Watanabe S, Ito T, Goto H, Wells K, McGregor M, Cooley AJ, Kawaoka Y (1999). Biological heterogeneity, including systemic replication in mice, of H5N1 influenza A virus isolates from humans in Hong Kong. J Virol.

[51] Gubareva LV, McCullers JA, Bethell RC, Webster RG (1998). Characterization of influenza A/HongKong/156/97 (H5N1) virus in a mouse model and protective effect of zanamivir on H5N1 infection in mice. J Infect Dis.

[52] Belser JA, Szretter KJ, Katz JM, Tumpey TM (2009). Use of animal models to understand the pandemic potential of highly pathogenic avian influenza viruses. Adv Virus Res.

[53] Belser JA, Lu X, Maines TR, Smith C, Li Y, Donis RO, Katz JM, Tumpey TM (2007). Pathogenesis of avian influenza (H7) virus infection in mice and ferrets: enhanced virulence of Eurasian H7N7 viruses isolated from humans. J Virol.

[54] Munster VJ, de Wit E, van Riel D, Beyer WE, Rimmelzwaan GF, Osterhaus AD, Kuiken T, Fouchier RA (2007). The molecular basis of the pathogenicity of the Dutch highly pathogenic human influenza A H7N7 viruses. J Infect Dis.

[55] Joseph T, McAuliffe J, Lu B, Jin H, Kemble G, Subbarao K (2007). Evaluation of replication and pathogenicity of avian influenza a H7 subtype viruses in a mouse model. J Virol.

[56] Driskell EA, Jones CA, Stallknecht DE, Howerth EW, Tompkins SM (2010). Avian influenza virus isolates from wild birds replicate and cause disease in a mouse model of infection. Virology.

[57] Belser JA, Wadford DA, Pappas C, Gustin KM, Maines TR, Pearce MB, Zeng H, Swayne DE, Pantin-Jackwood M, Katz JM (2010). Pathogenesis of pandemic influenza A (H1N1) and triple-reassortant swine influenza A (H1) viruses in mice. J Virol.

[58] Maines TR, Jayaraman A, Belser JA, Wadford DA, Pappas C, Zeng H, Gustin KM, Pearce MB, Viswanathan K, Shriver ZH (2009). Transmission and pathogenesis of swine-origin 2009 A(H1N1) influenza viruses in ferrets and mice. Science.

[59] Tumpey TM, Garcia-Sastre A, Mikulasova A, Taubenberger JK, Swayne DE, Palese P, Basler CF (2002). Existing antivirals are effective against influenza viruses with genes from the 1918 pandemic virus. Proc Natl Acad Sci U S A.

[60] Yen HL, Monto AS, Webster RG, Govorkova EA (2005). Virulence may determine the necessary duration and dosage of oseltamivir treatment for highly pathogenic A/Vietnam/1203/04 influenza virus in mice. J Infect Dis.

[61] Boltz DA, Ilyushina NA, Arnold CS, Babu YS, Webster RG, Govorkova EA (2008). Intramuscularly administered neuraminidase inhibitor peramivir is effective against lethal H5N1 influenza virus in mice. Antiviral Res.

[62] Govorkova EA, Ilyushina NA, McClaren JL, Naipospos TS, Douangngeun B, Webster RG (2009). Susceptibility of highly pathogenic H5N1 influenza viruses to the neuraminidase inhibitor oseltamivir differs *in vitro* and in a mouse model. Antimicrob Agents Chemother.

[63] Ilyushina NA, Hay A, Yilmaz N, Boon AC, Webster RG, Govorkova EA (2008). Oseltamivir-ribavirin combination therapy for highly pathogenic H5N1 influenza virus infection in mice. Antimicrob Agents Chemother.

[64] Ilyushina NA, Hoffmann E, Salomon R, Webster RG, Govorkova EA (2007). Amantadine-oseltamivir combination therapy for H5N1 influenza virus infection in mice. Antivir Ther.

[65] Govorkova EA, Leneva IA, Goloubeva OG, Bush K, Webster RG (2001). Comparison of efficacies of RWJ-270201, zanamivir, and oseltamivir against H5N1, H9N2, and other avian influenza viruses. Antimicrob Agents Chemother.

[66] Malakhov MP, Aschenbrenner LM, Smee DF, Wandersee MK, Sidwell RW, Gubareva LV, Mishin VP, Hayden FG, Kim DH, Ing A (2006). Sialidase Fusion Protein as a Novel Broad-Spectrum Inhibitor of Influenza Virus Infection. Antimicrob Agents Chemother.

[67] Prabhu N, Prabakaran M, Ho HT, Velumani S, Qiang J, Goutama M, Kwang J (2009). Monoclonal antibodies against the fusion peptide of hemagglutinin protect mice from lethal influenza A virus H5N1 infection. J Virol.

[68] Duan M, Zhou Z, Lin RX, Yang J, Xia XZ, Wang SQ (2008). *In vitro* and *in vivo* protection against the highly pathogenic H5N1 influenza virus by an antisense phosphorothioate oligonucleotide. Antivir Ther.

[69] Belser JA, Lu X, Szretter KJ, Jin X, Aschenbrenner LM, Lee A, Hawley S, Kim do H, Malakhov MP, Yu M (2007). DAS181, a novel sialidase fusion protein, protects mice from lethal avian influenza H5N1 virus infection. J Infect Dis.

[70] Kiso M, Takahashi K, Sakai-Tagawa Y, Shinya K, Sakabe S, Le QM, Ozawa M, Furuta Y, Kawaoka Y (2010). T-705 (favipiravir) activity against lethal H5N1 influenza A viruses. Proc Natl Acad Sci U S A.

[71] Smee DF, Hurst BL, Wong MH, Bailey KW, Tarbet EB, Morrey JD, Furuta Y (2010). Effects of the combination of favipiravir (T-705) and oseltamivir on influenza a virus infections in mice. Antimicrob Agents Chemother.

[72] Sidwell RW, Barnard DL, Day CW, Smee DF, Bailey KW, Wong MH, Morrey JD, Furuta Y (2007). Efficacy of orally administered T-705 on lethal avian influenza A (H5N1) virus infections in mice. Antimicrob Agents Chemother.

[73] Manicassamy B, Medina RA, Hai R, Tsibane T, Stertz S, Nistal-Villan E, Palese P, Basler CF, Garcia-Sastre A (2010). Protection of mice against lethal challenge with 2009 H1N1 influenza A virus by 1918-like and classical swine H1N1 based vaccines. PLoS Pathog.

[74] Castrucci MR, Kawaoka Y (1993). Biologic importance of neuraminidase stalk length in influenza A virus. J Virol.

[75] Kobasa D, Takada A, Shinya K, Hatta M, Halfmann P, Theriault S, Suzuki H, Nishimura H, Mitamura K, Sugaya N (2004). Enhanced virulence of influenza A viruses with the haemagglutinin of the 1918 pandemic virus. Nature.

[76] Maines TR, Lu XH, Erb SM, Edwards L, Guarner J, Greer PW, Nguyen DC, Szretter KJ, Chen LM, Thawatsupha P (2005). Avian influenza (H5N1) viruses isolated from humans in Asia in 2004 exhibit increased virulence in mammals. J Virol.

[77] Connor RJ, Kawaoka Y, Webster RG, Paulson JC (1994). Receptor Specificity in Human, Avian, and Equine H2 and H3 Influenza Virus Isolates. Virology.

[78] Rogers GN, D’Souza BL (1989). Receptor binding properties of human and animal H1 influenza virus isolates. Virology.

[79] Ibricevic A, Pekosz A, Walter MJ, Newby C, Battaile JT, Brown EG, Holtzman MJ, Brody SL (2006). Influenza virus receptor specificity and cell tropism in mouse and human airway epithelial cells. J Virol.

[80] Shinya K, Ebina M, Yamada S, Ono M, Kasai N, Kawaoka Y (2006). Avian flu: Influenza virus receptors in the human airway. Nature.

[81] van Riel D, Munster VJ, de Wit E, Rimmelzwaan GF, Fouchier RAM, Osterhaus ADME, Kuiken T (2007). Human and Avian Influenza Viruses Target Different Cells in the Lower Respiratory Tract of Humans and Other Mammals. Am J Pathol.

[82] Dybing JK, Schultz-Cherry S, Swayne DE, Suarez DL, Perdue ML (2000). Distinct pathogenesis of hong kong-origin H5N1 viruses in mice compared to that of other highly pathogenic H5 avian influenza viruses. J Virol.

[83] Garigliany MM, Habyarimana A, Lambrecht B, Van de Paar E, Cornet A, van den Berg T, Desmecht D (2010). Influenza A strain-dependent pathogenesis in fatal H1N1 and H5N1 subtype infections of mice. Emerg Infect Dis.

[84] Perrone LA, Plowden JK, Garcia-Sastre A, Katz JM, Tumpey TM (2008). H5N1 and 1918 pandemic influenza virus infection results in early and excessive infiltration of macrophages and neutrophils in the lungs of mice. PLoS Pathog.

[85] Wolk KE, Lazarowski ER, Traylor ZP, Yu EN, Jewell NA, Durbin RK, Durbin JE, Davis IC (2008). Influenza A virus inhibits alveolar fluid clearance in BALB/c mice. Am J Respir Crit Care Med.

[86] Jang H, Boltz D, Sturm-Ramirez K, Shepherd KR, Jiao Y, Webster R, Smeyne RJ (2009). Highly pathogenic H5N1 influenza virus can enter the central nervous system and induce neuroinflammation and neurodegeneration. Proc Natl Acad Sci U S A.

[87] Rowe T, Cho DS, Bright RA, Zitzow LA, Katz JM (2003). Neurological manifestations of avian influenza viruses in mammals. Avian Dis.

[88] Leyva-Grado VH, Churchill L, Harding J, Krueger JM (2010). The olfactory nerve has a role in the body temperature and brain cytokine responses to influenza virus. Brain Behav Immun.

[89] Majde JA, Bohnet SG, Ellis GA, Churchill L, Leyva-Grado V, Wu M, Szentirmai E, Rehman A, Krueger JM (2007). Detection of mouse-adapted human influenza virus in the olfactory bulbs of mice within hours after intranasal infection. J Neurovirol.

[90] Yang YT, Evans CA (1961). Hypothermia in mice due to influenza virus infection. Proc Soc Exp Biol Med.

[91] Hatta M, Hatta Y, Kim JH, Watanabe S, Shinya K, Nguyen T, Lien PS, Le QM, Kawaoka Y (2007). Growth of H5N1 influenza A viruses in the upper respiratory tracts of mice. PLoS Pathog.

[92] de Jong MD, Cam BV, Qui PT, Hien VM, Thanh TT, Hue NB, Beld M, Phuong LT, Khanh TH, Chau NVV (2005). Fatal Avian Influenza A (H5N1) in a Child Presenting with Diarrhea Followed by Coma. N Engl J Med.

[93] Sidwell RW, Huffman JH, Gilbert J, Moscon B, Pedersen G, Burger R, Warren RP (1992). Utilization of pulse oximetry for the study of the inhibitory effects of antiviral agents on influenza virus in mice. Antimicrob Agents Chemother.

[94] Sidwell RW, Huffman JH, Barnard DL, Bailey KW, Wong MH, Morrison A, Syndergaard T, Kim CU (1998). Inhibition of influenza virus infections in mice by GS4104, an orally effective influenza virus neuraminidase inhibitor. Antiviral Res.

[95] Droebner K, Ehrhardt C, Poetter A, Ludwig S, Planz O (2007). CYSTUS052, a polyphenol-rich plant extract, exerts anti-influenza virus activity in mice. Antiviral Res.

[96] Davies WL, Grunert RR, Haff RF, McGahen JW, Neumayer EM, Paulshock M, Watts JC, Wood TR, Hermann EC, Hoffmann CE (1964). Antiviral Activity of 1-Adamantanamine (Amantadine). Science.

[97] Grunert RR, McGahen JW, Davies WL (1965). The *in vivo* Antiviral Activity of 1-Adamantanamine (Amantadine). I. Prophylactic and Therapeutic Activity against Influenza Viruses. Virology.

[98] Rabinovich S (1972). Rimantadine therapy of influenza A infection in mice. Antimicrob Agents Chemother.

[99] Stephen EL, Dominik JW, Moe JB, Spertzel RO, Walker JS (1975). Treatment of influenza infection of mice by using rimantadine hydrochlorides by the aerosol and intraperitoneal routes. Antimicrob Agents Chemother.

[100] Mendel DB, Tai CY, Escarpe PA, Li W, Sidwell RW, Huffman JH, Sweet C, Jakeman KJ, Merson J, Lacy SA (1998). Oral administration of a prodrug of the influenza virus neuraminidase inhibitor GS 4071 protects mice and ferrets against influenza infection. Antimicrob Agents Chemother.

[101] von Itzstein M, Wu WY, Kok GB, Pegg MS, Dyason JC, Jin B, Van Phan T, Smythe ML, White HF, Oliver SW (1993). Rational design of potent sialidase-based inhibitors of influenza virus replication. Nature.

[102] Babu YS, Chand P, Bantia S, Kotian P, Dehghani A, El-Kattan Y, Lin TH, Hutchison TL, Elliott AJ, Parker CD (2000). BCX-1812 (RWJ-270201): discovery of a novel, highly potent, orally active, and selective influenza neuraminidase inhibitor through structure-based drug design. J Med Chem.

[103] Bantia S, Parker CD, Ananth SL, Horn LL, Andries K, Chand P, Kotian PL, Dehghani A, El-Kattan Y, Lin T (2001). Comparison of the anti-influenza virus activity of RWJ-270201 with those of oseltamivir and zanamivir. Antimicrob Agents Chemother.

[104] Sidwell RW, Smee DF, Huffman JH, Barnard DL, Bailey KW, Morrey JD, Babu YS (2001). *In vivo* influenza virus-inhibitory effects of the cyclopentane neuraminidase inhibitor RJW-270201. Antimicrob Agents Chemother.

[105] Schulman JL (1970). Effects of immunity on transmission of influenza: experimental studies. Prog Med Virol.

[106] Schulman JL (1967). Experimental transmission of influenza virus infection in mice. 3. Differing effects of immunity induced by infection and by inactivated influenza virus vaccine on transmission of infection. J Exp Med.

[107] Schulman JL (1967). Experimental transmission of influenza virus infection in mice: IV. Relationship of transmissibility of different strains of virus and recovery of airborne virus in the environment of infector mice. J Exp Med.

[108] Schulman JL (1968). The use of an animal model to study transmission of influenza virus infection. Am J Public Health Nations Health.

[109] Schulman JL, Kilbourne ED (1962). Airborne transmission of influenza virus infection in mice. Nature.

[110] Schulman JL, Kilbourne ED (1963). Experimental Transmission of Influenza Virus Infection in Mice. Ii. Some Factors Affecting the Incidence of Transmitted Infection. J Exp Med.

[111] Schulman JL, Kilbourne ED (1963). Experimental transmission of influenza virus infection in mice: I. The period of transmissibility. J Exp Med.

[112] Lowen AC, Mubareka S, Tumpey TM, García-Sastre A, Palese P (2006). The guinea pig as a transmission model for human influenza viruses. Proc Natl Acad Sci U S A.

[113] Francis T, Magill TP (1935). Immunological Studies with the Virus of Influenza. J Exp Med.

[114] Francis T, Stuart-Harris CH (1938). Studies on the Nasal Histology of Epidemic Influenza Virus Infection in the Ferret : Iii. Histological and Serological Observations on Ferrets Receiving Repeated Inoculations of Epidemic Infuenza Virus. J Exp Med.

[115] Francis T, Stuart-Harris CH (1938). Studies on the Nasal Histology of Epidemic Influenza Virus Infection in the Ferret : I. The Development and Repair of the Nasal Lesion. J Exp Med.

[116] Shope RE (1934). The Infection of Ferrets with Swine Influenza Virus. J Exp Med.

[117] Shope RE (1934). Swine Influenza : V. Studies on Contagion. J Exp Med.

[118] Shope RE (1935). The Infection of Mice with Swine Influenza Virus. J Exp Med.

[119] Smith W, Andrewes CH, Laidlaw PP (1933). A virus obtained from influenza patients. Lancet.

[120] Buchman CA, Swarts JD, Seroky JT, Panagiotou N, Hayden F, Doyle WJ (1995). Otologic and systemic manifestations of experimental influenza A virus infection in the ferret. Otolaryngol Head Neck Surg.

[121] Mubareka S, Lowen AC, Steel J, Coates AL, Garcia-Sastre A, Palese P (2009). Transmission of Influenza Virus via Aerosols and Fomites in the Guinea Pig Model. J Infect Dis.

[122] Sweet C, Bird RA, Coates DM, Overton HA, Smith H (1985). Recent H1N1 viruses (A/USSR/90/77, A/Fiji/15899/83, A/Firenze/13/83) replicate poorly in ferret bronchial epithelium. Brief report. Arch Virol.

[123] Baz M, Abed Y, Simon P, Hamelin ME, Boivin G (2010). Effect of the Neuraminidase Mutation H274Y Conferring Resistance to Oseltamivir on the Replicative Capacity and Virulence of Old and Recent Human Influenza A(H1N1) Viruses. J Infect Dis.

[124] Munster VJ, de Wit E, van den Brand JM, Herfst S, Schrauwen EJ, Bestebroer TM, van de Vijver D, Boucher CA, Koopmans M, Rimmelzwaan GF (2009). Pathogenesis and Transmission of Swine-Origin 2009 A(H1N1) Influenza Virus in Ferrets. Science.

[125] Herlocher ML, Elias S, Truscon R, Harrison S, Mindell D, Simon C, Monto AS (2001). Ferrets as a transmission model for influenza: sequence changes in HA1 of type A (H3N2) virus. J Infect Dis.

[126] Jackson S, Van Hoeven N, Chen LM, Maines TR, Cox NJ, Katz JM, Donis RO (2009). Reassortment between avian H5N1 and human H3N2 influenza viruses in ferrets: a public health risk assessment. J Virol.

[127] Round EM, Stebbing N (1981). Antiviral effects of single-stranded polynucleotide inhibitors of the influenza virion-associated transcriptase against influenza virus infection of hamsters and ferrets. Antiviral Res.

[128] Sweet C, Hayden FG, Jakeman KJ, Grambas S, Hay AJ (1991). Virulence of rimantadine-resistant human influenza A (H3N2) viruses in ferrets. J Infect Dis.

[129] Boltz DA, Rehg JE, McClaren J, Webster RG, Govorkova EA (2008). Oseltamivir prophylactic regimens prevent H5N1 influenza morbidity and mortality in a ferret model. J Infect Dis.

[130] Cameron CM, Cameron MJ, Bermejo-Martin JF, Ran L, Xu L, Turner PV, Ran R, Danesh A, Fang Y, Chan PK (2008). Gene expression analysis of host innate immune responses during Lethal H5N1 infection in ferrets. J Virol.

[131] Zitzow LA, Rowe T, Morken T, Shieh WJ, Zaki S, Katz JM (2002). Pathogenesis of avian influenza A (H5N1) viruses in ferrets. J Virol.

[132] Jakeman KJ, Tisdale M, Russell S, Leone A, Sweet C (1994). Efficacy of 2′-deoxy-2′-fluororibosides against influenza A and B viruses in ferrets. Antimicrob Agents Chemother.

[133] Kim YH, Kim HS, Cho SH, Seo SH (2009). Influenza B virus causes milder pathogenesis and weaker inflammatory responses in ferrets than influenza A virus. Viral Immunol.

[134] Mishin VP, Hayden FG, Gubareva LV (2005). Susceptibilities of antiviral-resistant influenza viruses to novel neuraminidase inhibitors. Antimicrob Agents Chemother.

[135] Belser JA, Blixt O, Chen LM, Pappas C, Maines TR, Van Hoeven N, Donis R, Busch J, McBride R, Paulson JC (2008). Contemporary North American influenza H7 viruses possess human receptor specificity: Implications for virus transmissibility. Proc Natl Acad Sci U S A.

[136] Wan H, Sorrell EM, Song H, Hossain MJ, Ramirez-Nieto G, Monne I, Stevens J, Cattoli G, Capua I, Chen LM (2008). Replication and transmission of H9N2 influenza viruses in ferrets: evaluation of pandemic potential. PLoS One.

[137] Francis T (1934). Transmission of Influenza by a Filterable Virus. Science.

[138] Call SA, Vollenweider MA, Hornung CA, Simel DL, McKinney WP (2005). Does this patient have influenza. JAMA.

[139] Eccles R (2002). An explanation for the seasonality of acute upper respiratory tract viral infections. Acta Otolaryngol.

[140] Hayden FG, Gubareva LV, Monto AS, Klein TC, Elliot MJ, Hammond JM, Sharp SJ, Ossi MJ (2000). Inhaled zanamivir for the prevention of influenza in families. Zanamivir Family Study Group. N Engl J Med.

[141] Matsuoka Y, Lamirande EW, Subbarao K (2009). The ferret model for influenza. Curr Protoc Microbiol.

[142] Maher JA, DeStefano J (2004). The ferret: an animal model to study influenza virus. Lab Anim (NY).

[143] Belshe RB, Smith MH, Hall CB, Betts R, Hay AJ (1988). Genetic basis of resistance to rimantadine emerging during treatment of influenza virus infection. J Virol.

[144] Tumpey TM, Maines TR, Van Hoeven N, Glaser L, Solorzano A, Pappas C, Cox NJ, Swayne DE, Palese P, Katz JM (2007). A two-amino acid change in the hemagglutinin of the 1918 influenza virus abolishes transmission. Science.

[145] van Riel D, Munster VJ, de Wit E, Rimmelzwaan GF, Fouchier RAM, Osterhaus ADME, Kuiken T (2006). H5N1 Virus Attachment to Lower Respiratory Tract. Science.

[146] van Riel D, Munster VJ, de Wit E, Rimmelzwaan GF, Fouchier RA, Osterhaus AD, Kuiken T (2007). Human and avian influenza viruses target different cells in the lower respiratory tract of humans and other mammals. Am J Pathol.

[147] Govorkova EA, Ilyushina NA, Boltz DA, Douglas A, Yilmaz N, Webster RG (2007). Efficacy of oseltamivir therapy in ferrets inoculated with different clades of H5N1 influenza virus. Antimicrob Agents Chemother.

[148] Yun NE, Linde NS, Zacks MA, Barr IG, Hurt AC, Smith JN, Dziuba N, Holbrook MR, Zhang L, Kilpatrick JM (2008). Injectable peramivir mitigates disease and promotes survival in ferrets and mice infected with the highly virulent influenza virus, A/Vietnam/1203/04 (H5N1). Virology.

[149] Roche Laboratories Inc. Tamiflu (Oseltamivir Phosphate) Capsules and for Oral Suspension, 2008. Available online: http://www.fda.gov/downloads/Drugs/DrugSafety/InformationbyDrugClass/UCM147992.pdf (accessed on 22 July 2010)

[150] Li W, Escarpe PA, Eisenberg EJ, Cundy KC, Sweet C, Jakeman KJ, Merson J, Lew W, Williams M, Zhang L (1998). Identification of GS 4104 as an orally bioavailable prodrug of the influenza virus neuraminidase inhibitor GS 4071. Antimicrob Agents Chemother.

[151] Cochran KW, Maassab HF, Tsunoda A, Berlin BS (1965). Studies on the antiviral activity of amantadine hydrochloride. Ann NY Acad Sci.

[152] Squires SL (1970). The evaluation of compounds against influenza viruses. Ann NY Acad Sci.

[153] Fenton RJ, Bessell C, Spilling CR, Potter CW (1977). The effects of peroral or local aerosol administration of 1-aminoadamantane hydrochloride (amantadine hydrochloride) on influenza infections of the ferret. J Antimicrob Chemother.

[154] Herlocher ML, Truscon R, Fenton R, Klimov A, Elias S, Ohmit SE, Monto AS (2003). Assessment of development of resistance to antivirals in the ferret model of influenza virus infection. J Infect Dis.

[155] Mendelman PM, Rappaport R, Cho I, Block S, Gruber W, August M, Dawson D, Cordova J, Kemble G, Mahmood K (2004). Live attenuated influenza vaccine induces cross-reactive antibody responses in children against an A/Fujian/411/2002-like H3N2 antigenic variant strain. Pediatr Infect Dis J.

[156] Maines TR, Chen LM, Matsuoka Y, Chen H, Rowe T, Ortin J, Falcon A, Nguyen TH, Mai le Q, Sedyaningsih ER (2006). Lack of transmission of H5N1 avian-human reassortant influenza viruses in a ferret model. Proc Natl Acad Sci U S A.

[157] Sorrell EM, Wan H, Araya Y, Song H, Perez DR (2009). Minimal molecular constraints for respiratory droplet transmission of an avian-human H9N2 influenza A virus. Proc Natl Acad Sci U S A.

[158] Yen HL, Herlocher LM, Hoffmann E, Matrosovich MN, Monto AS, Webster RG, Govorkova EA (2005). Neuraminidase inhibitor-resistant influenza viruses may differ substantially in fitness and transmissibility. Antimicrob Agents Chemother.

[159] Lowen AC, Mubareka S, Steel J, Palese P (2007). Influenza virus transmission is dependent on relative humidity and temperature. PLoS Pathog.

[160] Lowen AC, Steel J, Mubareka S, Carnero E, Garcia-Sastre A, Palese P (2009). Blocking inter-host transmission of influenza virus by vaccination in the guinea pig model. J Virol.

[161] Lowen AC, Steel J, Mubareka S, Palese P (2008). High temperature (30°C) blocks aerosol but not contact transmission of influenza virus. J Virol.

[162] Phair JP, Kauffman CA, Jennings R, Potter CW (1979). Influenza virus infection of the guinea pig: immune response and resistance. Med Microbiol Immunol.

[163] Tang X, Chong KT (2009). Histopathology and growth kinetics of influenza viruses (H1N1 and H3N2) in the upper and lower airways of guinea pigs. J Gen Virol.

[164] Van Hoeven N, Belser JA, Szretter KJ, Zeng H, Staeheli P, Swayne DE, Katz JM, Tumpey TM (2009). Pathogenesis of the 1918 pandemic and H5N1 influenza virus infection in a guinea pig model: The antiviral potential of exogenous alpha-interferon to reduce virus shedding. J Virol.

[165] Steel J, Staeheli P, Mubareka S, Garcia-Sastre A, Palese P, Lowen AC (2010). Transmission of pandemic H1N1 influenza virus and impact of prior exposure to seasonal strains or interferon treatment. J Virol.

[166] Kwon YK, Lipatov AS, Swayne DE (2009). Bronchointerstitial Pneumonia in Guinea Pigs Following Inoculation with H5N1 High Pathogenicity Avian Influenza Virus. Vet Pathol.

[167] Steel J, Lowen AC, Mubareka S, Palese P (2009). Transmission of influenza virus in a mammalian host is increased by PB2 amino acids 627K or 627E/701N. PLoS Pathog.

[168] Gao Y, Zhang Y, Shinya K, Deng G, Jiang Y, Li Z, Guan Y, Tian G, Li Y, Shi J (2009). Identification of amino acids in HA and PB2 critical for the transmission of H5N1 avian influenza viruses in a mammalian host. PLoS Pathog.

[169] Bouvier NM, Lowen AC, Palese P (2008). Oseltamivir-resistant influenza A viruses are transmitted efficiently among guinea pigs by direct contact but not by aerosol. J Virol.

[170] Lowen AC, Palese P (2008).

[171] Azoulay-Dupuis E, Lambre CR, Soler P, Moreau J, Thibon M (1984). Lung alterations in guinea-pigs infected with influenza virus. J Comp Path.

[172] Steel J, Lowen AC, Palese P (2009).

[173] Ottolini MG, Blanco JC, Eichelberger MC, Porter DD, Pletneva L, Richardson JY, Prince GA (2005). The cotton rat provides a useful small-animal model for the study of influenza virus pathogenesis. J Gen Virol.

[174] Eichelberger MC, Prince GA, Ottolini MG (2004). Influenza-induced tachypnea is prevented in immune cotton rats, but cannot be treated with an anti-inflammatory steroid or a neuraminidase inhibitor. Virology.

[175] Ottolini M, Blanco J, Porter D, Peterson L, Curtis S, Prince G (2003). Combination anti-inflammatory and antiviral therapy of influenza in a cotton rat model. Pediatr Pulmonol.

[176] Straight TM, Ottolini MG, Prince GA, Eichelberger MC (2008). Antibody contributes to heterosubtypic protection against influenza A-induced tachypnea in cotton rats. Virol J.

[177] Straight TM, Ottolini MG, Prince GA, Eichelberger MC (2006). Evidence of a cross-protective immune response to influenza A in the cotton rat model. Vaccine.

[178] Taylor RM, Parodi AS (1942). Use of hamster (Cricetus auratus) for the detection of influenza virus in throat washings. Proc Soc Exp Biol Med.

[179] Ali MJ, Teh CZ, Jennings R, Potter CW (1982). Transmissibility of influenza viruses in hamsters. Arch Virol.

[180] Jennings R, Potter CW, McLaren C (1974). Effect of preinfection and preimmunization on the serum antibody response to subsequent immunization with heterotypic influenza vaccines. J Immunol.

[181] Potter CW, Jennings R (1976). The hamster as a model system for the study of influenza vaccines. Postgrad Med J.

[182] Mills J, Chanock V, Chanock RM (1971). Temperature-sensitive mutants of influenza virus. I. Behavior in tissue culture and in experimental animals. J Infect Dis.

[183] Abou-Donia H, Jennings R, Potter CW (1980). Growth of influenza A viruses in hamsters. Arch Virol.

[184] Heath AW, Addison C, Ali M, Teale D, Potter CW (1983). *In vivo* and *in vitro* hamster models in the assessment of virulence of recombinant influenza viruses. Antiviral Res.

[185] Reeve P (1978). Growth of some attenuated influenza A viruses in hamsters. Med Microbiol Immunol.

[186] Reeve P, Pibermann M, Gerendas B (1981). Studies with some influenza B viruses in cell cultures, hamsters and hamster tracheal organ cultures. Med Microbiol Immunol.

[187] Baas T, Baskin CR, Diamond DL, Garcia-Sastre A, Bielefeldt-Ohmann H, Tumpey TM, Thomas MJ, Carter VS, Teal TH, Van Hoeven N (2006). Integrated molecular signature of disease: analysis of influenza virus-infected macaques through functional genomics and proteomics. J Virol.

[188] Baskin CR, Bielefeldt-Ohmann H, Garcia-Sastre A, Tumpey TM, Van Hoeven N, Carter VS, Thomas MJ, Proll S, Solorzano A, Billharz R (2007). Functional genomic and serological analysis of the protective immune response resulting from vaccination of macaques with an NS1-truncated influenza virus. J Virol.

[189] Carroll TD, Matzinger SR, Genesca M, Fritts L, Colon R, McChesney MB, Miller CJ (2008). Interferon-induced expression of MxA in the respiratory tract of rhesus macaques is suppressed by influenza virus replication. J Immunol.

[190] Fan J, Liang X, Horton MS, Perry HC, Citron MP, Heidecker GJ, Fu TM, Joyce J, Przysiecki CT, Keller PM (2004). Preclinical study of influenza virus A M2 peptide conjugate vaccines in mice, ferrets, and rhesus monkeys. Vaccine.

[191] Kobasa D, Jones SM, Shinya K, Kash JC, Copps J, Ebihara H, Hatta Y, Kim JH, Halfmann P, Hatta M (2007). Aberrant innate immune response in lethal infection of macaques with the 1918 influenza virus. Nature.

[192] Saslaw S, Wilson HE, Doan CA, Woolpert OC, Schwab JL (1946). Reactions of Monkeys to Experimentally Induced Influenza Virus a Infection : An Analysis of the Relative Roles of Humoral and Cellular Immunity under Conditions of Optimal or Deficient Nutrition. J Exp Med.

[193] Cilloniz C, Shinya K, Peng X, Korth MJ, Proll SC, Aicher LD, Carter VS, Chang JH, Kobasa D, Feldmann F (2009). Lethal influenza virus infection in macaques is associated with early dysregulation of inflammatory related genes. PLoS Pathog.

[194] Rimmelzwaan G, Baars M, van Amerongen G, van Beek R, Osterhaus ADME (2002). A single dose of an ISCOM influenza vaccine induces long-lasting protective immunity against homologous challenge infection but fails to protect Cynomolgus macaques against distant drift variants of influenza A (H3N2) viruses. Vaccine.

[195] Fan S, Gao Y, Shinya K, Li CK, Li Y, Shi J, Jiang Y, Suo Y, Tong T, Zhong G (2009). Immunogenicity and protective efficacy of a live attenuated H5N1 vaccine in nonhuman primates. PLoS Pathog.

[196] Kreijtz JH, Bodewes R, van Amerongen G, Kuiken T, Fouchier RA, Osterhaus AD, Rimmelzwaan GF (2007). Primary influenza A virus infection induces cross-protective immunity against a lethal infection with a heterosubtypic virus strain in mice. Vaccine.

[197] Laddy DJ, Yan J, Khan AS, Andersen H, Cohn A, Greenhouse J, Lewis M, Manischewitz J, King LR, Golding H (2009). Electroporation of synthetic DNA antigens offers protection in nonhuman primates challenged with highly pathogenic avian influenza virus. J Virol.

[198] Rimmelzwaan GF, Kuiken T, van Amerongen G, Bestebroer TM, Fouchier RA, Osterhaus AD (2001). Pathogenesis of influenza A (H5N1) virus infection in a primate model. J Virol.

[199] Rimmelzwaan GF, Kuiken T, van Amerongen G, Bestebroer TM, Fouchier RA, Osterhaus AD (2003). A primate model to study the pathogenesis of influenza A (H5N1) virus infection. Avian Dis.

[200] Stittelaar KJ, Tisdale M, van Amerongen G, van Lavieren RF, Pistoor F, Simon J, Osterhaus AD (2008). Evaluation of intravenous zanamivir against experimental influenza A (H5N1) virus infection in cynomolgus macaques. Antiviral Res.

[201] Ferko B, Stasakova J, Romanova J, Kittel C, Sereinig S, Katinger H, Egorov A (2004). Immunogenicity and protection efficacy of replication-deficient influenza A viruses with altered NS1 genes. J Virol.

